# Comparative efficacy of vasoactive medications in patients with septic shock: a network meta-analysis of randomized controlled trials

**DOI:** 10.1186/s13054-019-2427-4

**Published:** 2019-05-14

**Authors:** Lu Cheng, Jing Yan, Shutang Han, Qiuhua Chen, Mingqi Chen, Hua Jiang, Jun Lu

**Affiliations:** 1 Department of Intensive Care Unit, Affiliated Hospital of Nanjing University of Chinese Medicine, 155 Hanzhong Road, Nanjing, 210029 China; 2 Key Laboratory for Metabolic Diseases in Chinese Medicine, First Clinical Medical College, Nanjing University of Chinese Medicine, 138 Xianlin Avenue, Nanjing, 210013 China; 3 Department of Center of Gastrointestinal Endoscopy, Affiliated Hospital of Nanjing University of Chinese Medicine, 155 Hanzhong Road, Nanjing, 210029 China

**Keywords:** Sepsis, Septic shock, Vasoactive agent, Hemodynamic, Norepinephrine, Terlipressin, Dopamine, Vasopressin

## Abstract

**Background:**

Catecholamines, especially norepinephrine, are the most frequently used vasopressors for treating patients with septic shock. During the recent decades, terlipressin, vasopressin V1A agonist, and even Ca^2+^ sensitizer were increasingly used by physicians. The aim of this study is to compare the efficacy of such different kinds of vasoactive medications on mortality among patients with septic shock.

**Methods:**

Relevant randomized controlled trials were identified by searching PubMed, Embase, Web of Science, and the Cochrane Central Register of Controlled Trials updated to February 22, 2018. A network meta-analysis was performed to evaluate the effect of different types of vasoactive medications. The primary outcome was 28-day mortality. Intensive care unit (ICU) mortality, hospital and ICU length of stay (LOS), and adverse events were also assessed.

**Results:**

A total of 43 trials with 5767 patients assessing 17 treatment modalities were included. Treatments ranking based on surface under the cumulative ranking curve values from largest to smallest were NE/DB 85.9%, TP 75.1%, NE/EP 74.6%, PI 74.1%, EP 72.5%, VP 66.1%, NE 59.8%, PE 53.0%, DA 42.1%, DX 38.2%, SP 27.0%, PA 24.3%, EX 22.8%, LE 21.5%, and DB 13.3% for 28-day mortality. Treatments ranking for ICU mortality were TP/NE 86.4%, TP 80.3%, TP/DB/NE 65.7%, VP/NE 62.8%, NE 57.4%, VP 56.5%, PE 48.4%, DA 33.0%, PA 27.5%, LE 22.1%, and DB 9.9%. The incidence of myocardial infarction was reported with NE/EP 3.33% (*n* = 1 of 30), followed by EP 3.11% (*n* = 5 of 161), and then VP 3.10% (*n* = 19 of 613), NE 3.03% (*n* = 43 of 1417), DA 2.21% (n = 19 of 858), NE/DB 2.01% (*n* = 4 of 199), LE 1.16% (*n* = 3 of 258), and PA 0.39% (*n* = 1 of 257). The incidence of arrhythmia was reported with DA 26.01% (*n* = 258 of 992), followed by EP 22.98% (*n* = 37 of 161), and then NE/DB 20.60% (*n* = 41 of 199), NE/EP 20.0% (*n* = 6 of 30), NE 8.33% (*n* = 127 of 1525), LE 5.81% (*n* = 15 of 258), PA 2.33% (*n* = 6 of 257), and VP 1.67% (*n* = 10 of 600).

**Conclusions:**

The use of norepinephrine plus dobutamine was associated with lower 28-day mortality for septic shock, especially among patients with lower cardiac output.

**Electronic supplementary material:**

The online version of this article (10.1186/s13054-019-2427-4) contains supplementary material, which is available to authorized users.

## Background

Sepsis is a life-threatening organ dysfunction caused by dysregulated host response to infection, while septic shock is a subset of sepsis with circulatory and cellular/metabolic dysfunction associated with a higher risk of mortality [1, 2]. Septic shock is a major healthcare problem that contributes to the most common causes of death in the intensive care unit (ICU) [3]. Septic shock is a distributive shock with decreased systemic vascular resistance and mean arterial pressure (MAP) [4]. If hemodynamic status is not maintained, mortality rates for septic shock may even reach 50% [5]. Management of septic shock involves treating the underlying cause, fluid resuscitation, and infusion of vasoactive medications to achieve the goal of increased perfusion blood pressure. The vasoactive medications may increase vascular resistance and raise MAP and perfusion in tissues and organs [6].

Current guidelines recommend the use of norepinephrine as the first-line vasopressor with intent to target MAP [7]. Adrenergic agonists and vasopressin analogs are used most commonly by physicians. Some new drugs were used increasingly during the last decades. Selepressin was even considered to be able to replace norepinephrine while maintaining adequate MAP, improving fluid balance and shorten the mechanical ventilation time [8]. To date, there were no studies that synthetically compared the effects of all these vasoactive medications. Methodologically, existing studies only pooled the direct comparisons between the two vasoactive medications [9, 10].

A network meta-analysis (NMA) was used to combine direct within-trial between-drug comparisons with indirect evidence from the other trials [11]. In this study, NMA was performed to update current clinical study data and determine the association among treatments with catecholamine, vasopressin, inotropic drug, and other vasoactive medications on mortality, length of stay (LOS), and adverse events.

## Methods

The study protocol was registered in the International Prospective Register of Systematic Reviews (CRD42018090437). The presentation of this study followed the Preferred Reporting Items for Systematic reviews and Meta-Analyses guidelines for reporting NMA [12].

### Patients

Patients with septic shock who received vasoactive medications were enrolled in this network meta-analysis.

### Interventions and comparisons

The interventions of interest were specified with vasoactive medications, irrespective of dose, duration, or co-intervention. The vasoactive medications were compared with an inactive control intervention (e.g., placebo or standard care), or with an active control intervention (e.g., other vasoactive medications or combination therapy with vasoactive medications).

### Outcomes

The primary outcome was death from any cause and was assessed 28 days after the start of infusions (28-day mortality). The mortality at 28 to 30 days was considered equivalent to 28-day mortality. Death between the start of infusions and when the patients were discharged from the ICU (ICU mortality) was also assessed. Secondary outcomes were ICU and hospital length of stay. Length of stay in the ICU and hospital was defined as the time spent in the ICU or hospital, respectively, during the index hospitalization. The adverse events were also evaluated and defined as any undesirable outcomes that included myocardial infarction, arrhythmia, or peripheral ischemia.

.

### Setting

Randomized controlled trials (RCTs) were included, irrespective of the publication date, setting, and risk of bias. Studies with English and Chinese languages were both included.

### Search strategy

Studies were identified by electronically searching MEDLINE, Embase, Web of Science and the Cochrane Central Register for Controlled Trials (CENTRAL), using search terms describing the study design, intervention, or comparator updated to February 22, 2018. The specific search strategy in MEDLINE is presented in Additional file 1. Hand searching the references of eligible studies was performed and consulted to identify additional trials.

### Data collection and extraction

The study data were collected and extracted using a standardized form. Two authors independently screened the titles and abstracts for eligibility. Full papers were assessed to confirm disagreement in existence according to the exclusion criteria by the two authors who recorded the main reason for the exclusion. Any disagreement was resolved by a third author through consensus.

The inclusion criteria included (1) patients who were definitely diagnosed with septic shock, (2) studies that compared the effect of vasoactive medications, and (3) randomized controlled trial studies.

The exclusion criteria were (1) non-relevant intervention, (2) studies that failed to acquire outcomes of interest, (3) non-adult studies, and (5) non-RCT such as review, letter, before and after study, observational study, case-control study, and case report.

### Assessment of risk of bias

The Cochrane Collaboration Tool was used to assess the risk of bias for the included studies [13]. The risk of bias was evaluated using the following domains: (1) random sequence generation, (2) allocation concealment, (3) blinding of participants and personnel, (4) blinding of outcome assessment, (5) incomplete outcome data, (6) selective reporting, and (7) other bias. Judgment as “low,” “unclear,” or “high” risk of bias was provided in each of the domains for each study. Studies with low risk of bias for all the domains were considered to be at low risk of bias. Studies with high risk of bias for one or more domains were considered to be at high risk of bias. Two authors assessed the performance and detection bias separately. The disagreements were resolved by discussion.

### Statistical analysis

Odds ratio (OR) with 95% confidence interval (CI) was used to calculate the difference for dichotomous outcomes, while standardized mean difference (SMD) with 95% CI was used for continuous variables. If the studies only reported the median and measure of dispersion, the data were converted to mean and standard deviation assuming a normal distribution, by using two simple formulae [14].

Clinical and methodological heterogeneity were assessed according to the study characteristics. Statistical heterogeneity among direct comparisons of the included trials was assessed by using the fixed-effects model with Mantel-Haenszel weighting, because some comparisons were expected to show heterogeneity [15]. Sensitivity analysis was conducted by sequentially omitting one study each time, to identify the potential influence on whether there was a significant heterogeneity among the included studies.

Transitivity assumption is a key assumption in network meta-analysis. In our study, norepinephrine was used as a reference treatment in all analyses. For each intervention, the estimate versus norepinephrine was synthesized to get an overall summary estimate, and a hierarchy of interventions based on the overall estimate was obtained. The pooled effect size for each intervention was synthesized to obtain an overall weighted average using inverse variance as weights. The assumption of consistency implies that the direct and indirect evidence were in statistical agreement for every pairwise comparison in a network. The local inconsistency was evaluated by using the loop-specific approach, while the “design-by-treatment” model was used to describe and check the assumption of inconsistency in the entire network for each outcome [16, 17]. Contribution plot was performed to evaluate the effect that each trial contributed to the NMA. The contribution of a study to the direct estimate is the percentage of information that comes from a specific study in the estimation of a direct relative effect using standard pairwise meta-analysis. For studies in which patients crossed over to another treatment, the analysis was still according to the first assigned group. The potential publication bias was evaluated by using funnel plots.

The key NMA technique is associated with evaluating the assumption underlying the statistical synthesis of direct and indirect evidence [18]. The surface under the cumulative ranking curve (SUCRA) values and rankograms were used to present the hierarchy of interventions for each outcome. SUCRA values show the percentage of effectiveness each intervention achieves compared to a hypothetical best intervention, which is always the best without uncertainty [19]. Generally, SUCRA values are interpreted as probabilities, and the larger the probability, the better the treatment.

The modified Grades of Recommendation, Assessment, Development and Evaluation (GRADE) tool for NMAs was used to evaluate the quality of evidence [20]. The quality results were classified as follows: (1) high quality—further research is very unlikely to change the confidence in the estimated effect; (2) moderate quality—further research is likely to have an important impact on the confidence in the estimated effect and may change the estimate; (3) low quality—further research is very likely to have an important impact on the confidence in the estimated effect and is likely to change the estimate; and (4) very low quality—where any estimated effect is highly uncertain [21].

The results were considered statistically significant at two-sided *P* value less than 0.05. All statistical analyses were performed using STATA 14.0 software (StataCorp, College Station, TX, USA).

## Results

**Fig. 1:**
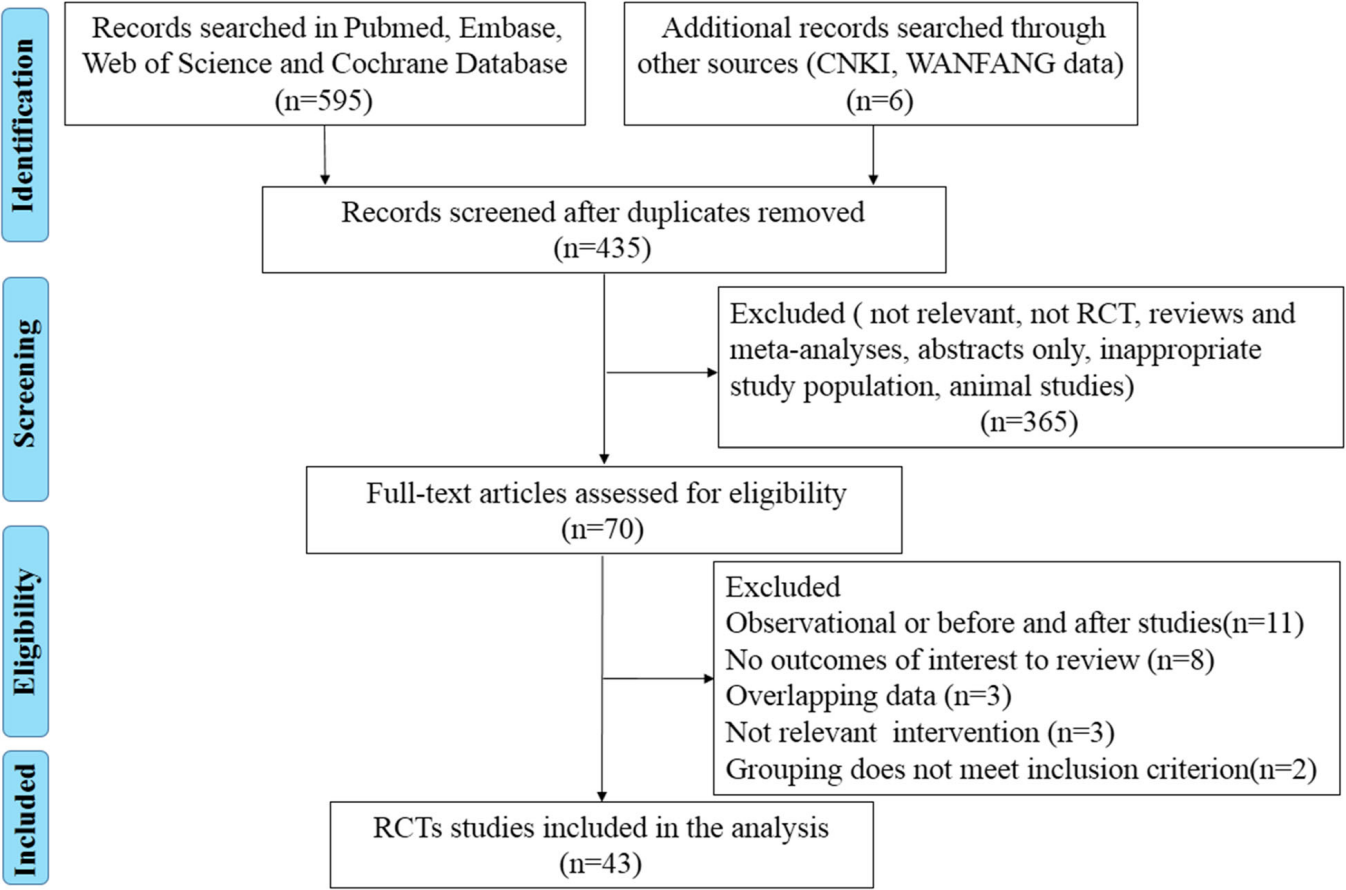
Flow chart depicting the process of identification of studies

A total of 601 studies from electronic databases were identified, and 166 duplicated studies were removed. After an initial evaluation of the titles and abstracts, 365 studies were excluded because they did not meet the predefined inclusion criteria. The remaining 70 studies were identified for full review, and 27 studies were excluded due to inappropriate study design, lack of outcomes of interest to review, and other reasons. Eventually, 43 randomized controlled trials with 5767 septic shock patients were included. The included studies were presented in Additional file 1. The flow diagram for the study inclusion according to PRISMA is shown in Fig. 1. These trials were conducted in 17 different countries, with Italy contributing the most (7 trials, 16.3%). The studies were published in English or Chinese. A total of 17 different interventions were identified in papers published between 1993 and 2017. The designs of the included RCTs were presented in Table 1.

**Table 1:** Studies designs of included randomized controlled trials

ID	Source	Country	Setting	Male/female	Treatment 1	Treatment 2	Treatment 3	Primary outcome
1	Martin et al. 1993 [32]	France	SC	24/8	Dopamine	Norepinephrine		Hemodynamics status
2	Marik and Mohedin 1994 [33]	USA	SC	11/9	Norepinephrine	Dopamine		Systemic and splanchnic oxygen utilization
3	Levy et al. 1997 [34]	France	SC	21/9	Epinephrine	Norepinephrine/dobutamine		Hemodynamics, lactate metabolism, and gastric tonometric variables
4	Malay et al. 1999 [35]	USA	SC	8/2	Vasopressin	Placebo		Arterial pressure
5	Kern et al. 2001 [36]	Germany	SC	NA	Enoximone	Dobutamine		Hepatosplanchnic function
6	Seguin et al. 2002 [37]	France	SC	12/10	Epinephrine	Norepinephrine/dobutamine		Systemic and pulmonary hemodynamics
7	Patel et al. 2002 [38]	Canada	MC	18/6	Norepinephrine	Vasopressin		Renal function
8	Dünser et al. 2003 [39]	Australia	SC	NA	Arginine vasopressin	Norepinephrine		Differences in hemodynamics
9	Morelli et al. 2005 [40]	Italy	MC	21/7	Dobutamine	Levosimendan		Systemic and regional hemodynamics
10	Albanèse et al. 2005 [41]	France	SC	13/7	Norepinephrine	Terlipressin		MAP
11	Luckner et al. 2006 [42]	Australia	SC	11/7	Vasopressin/norepinephrine	Norepinephrine		Cutaneous vascular reactivity
12	Seguin et al. 2006 [43]	France	SC	17/5	Dopexamine/norepinephrine	Epinephrine		Gastric perfusion
13	Schmoelz et al. 2006 [44]	Germany	SC	35/26	Dopexamine	Dopamine	Placebo	Systemic and renal effects
14	Lauzier et al. 2006 [45]	Canada, France	MC	14/9	Vasopressin	Norepinephrine		Hemodynamic parameters and SOFA score
15	Mathur et al. 2007 [46]	India	SC	32/18	Dopamine	Norepinephrine		Hemodynamic parameters
16	Annane et al. 2007 [47]	France	MC	202/128	Epinephrine	Norepinephrine/dobutamine		28-day all-cause mortality
17	Myburgh et al. 2008 [48]	Australia	MC	167/110	Epinephrine	Norepinephrine		The time taken to achieve a clinician-prescribed MAP goal
18	Morelli et al. 2008 [49]	Italy	SC	43/16	Norepinephrine	Terlipressin/norepinephrine	Terlipressin/dobutamine/norepinephrine	Systemic, pulmonary, and regional hemodynamic measurements and blood gases
19	Morelli et al. 2008 [50]	Italy	SC	21/11	Norepinephrine	Phenylephrine		Hemodynamic parameters
20	Russell et al. 2008 [51]	Canada, Australia, USA	MC	475/304	Norepinephrine	Vasopressin		Death from any cause
21	Morelli et al. 2009 [52]	Italy	SC	21/9	Terlipressin	Norepinephrine	Vasopressin	Systemic and regional hemodynamics
22	Alhashemi et al. 2009 [53]	Saudi Arabia	SC	NA	Levosimendan	Dobutamine		Central venous saturation (ScvO2) and serum lactate
23	De Backer et al. 2010 [54]	Belgium, Austria, Spain	MC	956/723	Dopamine	Norepinephrine		The rate of death at 28 days
24	Jain and Singh 2010 [55]	India	SC	28/26	Norepinephrine	Phenylephrine		Hemodynamic parameters
25	Patel et al. 2010 [56]	USA	SC	116/136	Dopamine	Norepinephrine		All-cause 28-day mortality
26	Morelli et al. 2010 [57]	Italy	SC	27/13	Levosimendan	Dobutamine		Systemic and microvascular hemodynamics
27	Morelli et al. 2011 [58]	Italy	SC	37/23	Terlipressin	Arginine vasopressin	Control	Microcirculatory perfusion
28	Han et al. 2012 [59]	China	MC	99/40	Pituitrin	Norepinephrine		The rate of death at 28 days
29	Mahmoud and Ammar 2012 [60]	Egypt	SC	31/29	Norepinephrine/dobutamine	Norepinephrine/epinephrine		SOFA score and cardiovascular effects
30	Memis et al. 2012 [61]	Turkey	SC	16/14	Dobutamine	Levosimendan		Regional blood flow
31	Mehta et al. 2013 [62]	Canada	MC	85/36	Norepinephrine	Vasopressin		Cardiac biomarkers and electrocardiograms
32	Hua et al. 2013 [63]	China	SC	18/14	Terlipressin	Dopamine		Hemodynamics and oxygenation variables
33	Fang and Dong 2014 [64]	China	SC	27/9	Dobutamine	Levosimendan		Hemodynamics and cardiac function
34	Torraco et al. 2014 [65]	Italy	SC	19/7	Levosimendan	Control		Mitochondrial function
35	Gordon et al. 2016 [66]	UK	MC	238/171	Vasopressin	Norepinephrine		Kidney failure-free days during the 28-day period
36	Xiao et al. 2016 [67]	China	SC	22/10	Norepinephrine	Terlipressin/norepinephrine		Tissue blood flow and organ function
37	Gordon et al. 2016 [68]	UK	MC	289/226	Levosimendan	Placebo		Mean daily SOFA score
38	Meng et al. 2016 [69]	China	SC	24/14	Levosimendan	Dobutamine		Biomarkers of myocardial injury and systemic hemodynamics
39	Barzegar et al. 2016 [70]	Iran	SC	19/11	Norepinephrine	Vasopressin		Lactate level and lactate clearance
40	Choudhury et al. 2016 [71]	India	SC	69/15	Terlipressin	Noradrenaline		Hemodynamics
41	Chen et al. 2017 [72]	China	SC	29/28	Norepinephrine	Terlipressin		Hemodynamics, volume responsiveness
42	Hajjej et al. 2017 [73]	Tunisia	SC	17/3	Levosimendan	Placebo		Cellular metabolism
43	Russell et al. 2017 [8]	Belgium, Denmark, USA	MC	27/21	Selepressin	Placebo		Stabilization of MAP as determined by the proportion of patients maintaining a MAP > 60 mmHg

The detailed management description and baseline characteristics of these trials were presented in Additional file 1. Norepinephrine was used most frequently in 23 trials. Vasopressin and levosimendan were both used in 9 trials. Dobutamine and dopamine were used in 7 and 6 trials, respectively, and epinephrine was used in 4 trials, while enoximone, pituitrin, and selepressin were all used in 1 trial. The combination of norepinephrine and dopamine was used in 4 trials, while the combinations of vasopressin and norepinephrine, epinephrine and norepinephrine, and terlipressin, dobutamine, and norepinephrine were used in 1 trial.

### Risk of bias

**Fig. 2:**
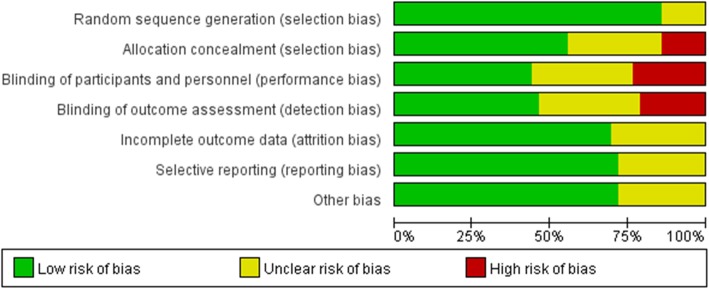
Risk of bias assessment: overall risk of bias for all included trials

As shown in Fig. 2, the most common risk was blinding of participants and personnel almost with one fourth of trials considered to be at high risk for bias. This was probably explained by the difficulty of blinding personnel who were performing 1 or more different medications in each patient. The lowest risk was random sequence generation, with exceeding 80% of trials considered to be at low risk for bias. The risk for random sequence generation, incomplete outcome data, selective reporting, and other bias was considered to be at high risk in non-trials. The details for the risk of bias were presented in (Additional file 1: Table S2). In summary, 24 trials were considered to be at low risk, and 14 trials were considered to be at unclear risk, while 5 trials were considered to be at high risk.

### Synthesis of results

Heterogeneity assessment, network geometry, SUCRA, forest plot, contribution plot, inconsistency analyses, and publication bias analyses were presented for most outcomes.

### Mortality

#### Twenty-eight-day mortality

**Fig. 3:**
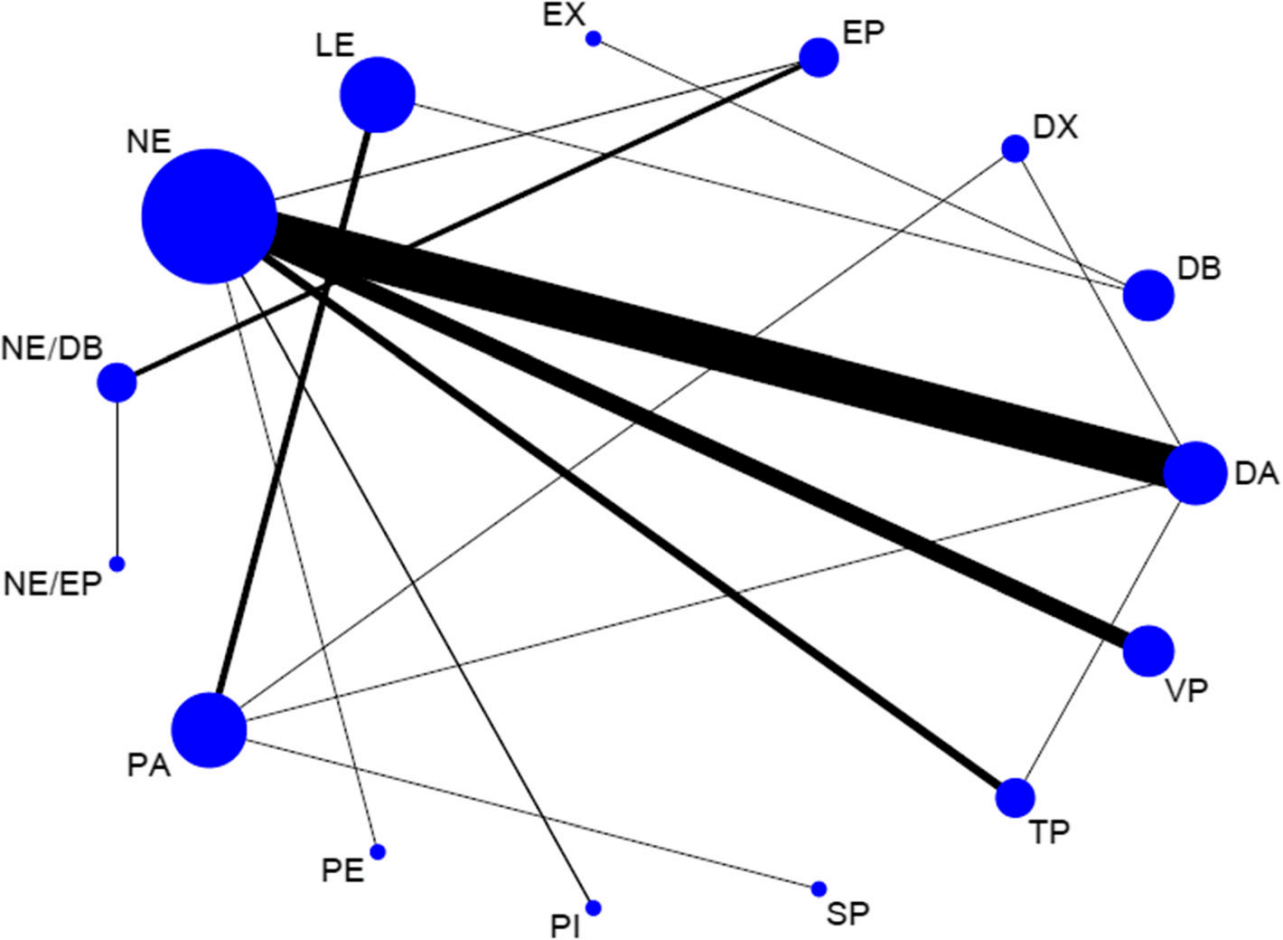
Network geometry. Network of all the included treatment agents for evaluating mortality. The size of the nodes was proportional to the number of patients randomized to each modality and thickness of the lines to the number of direct comparisons. For example, the circle area for NE was the largest, and the edge between NE and DA was much wider, indicating that the number of studies on NE was the highest, and the direct comparisons between NE and DA were the most common in the existed literatures

**Fig. 4:**
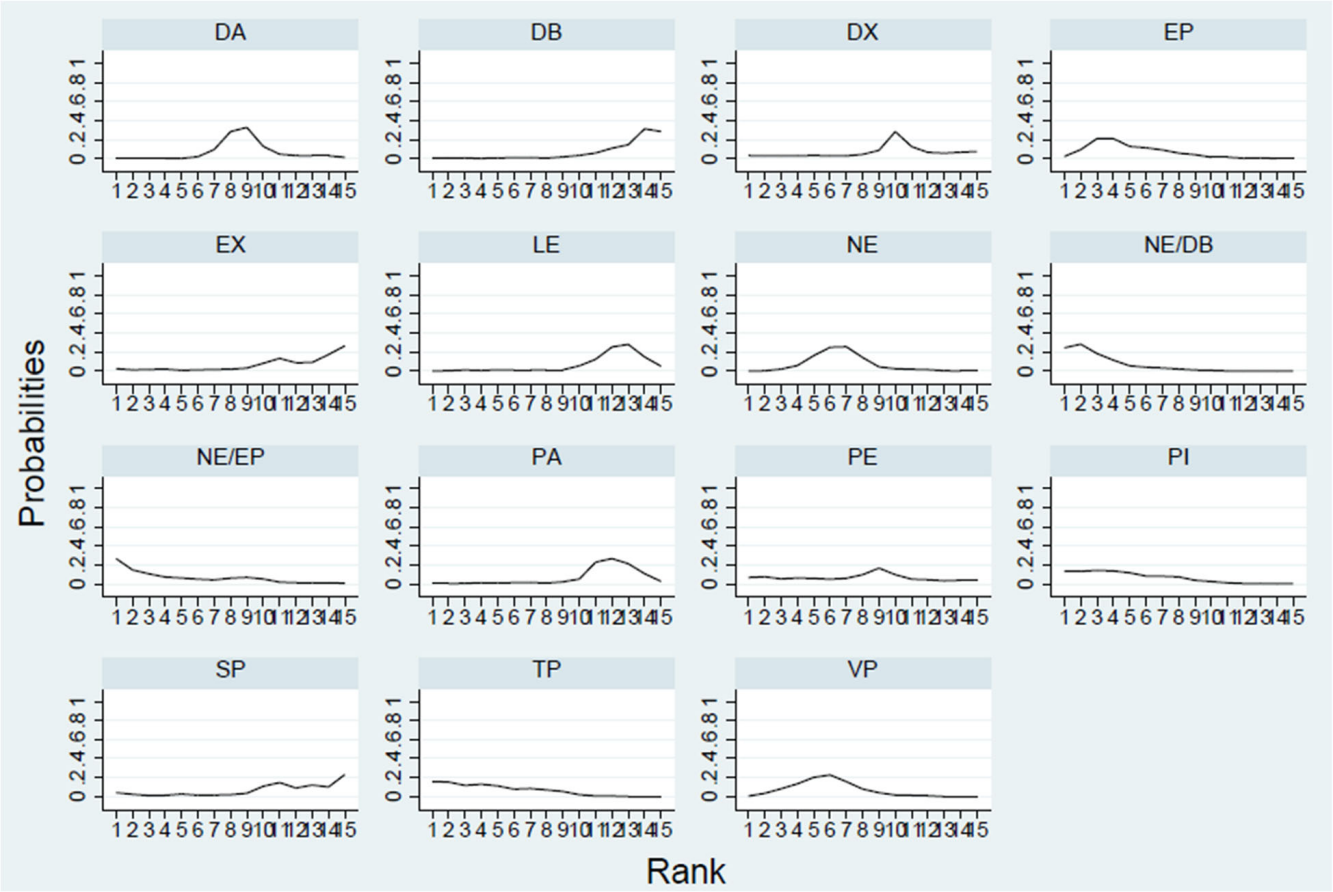
Twenty-eight-day mortality ranking among different interventions. A simple numerical summary to present the graphical display of cumulative ranking was used to estimate the surface under the cumulative ranking (SUCRA) line for each treatment. SUCRA would be 100% when a treatment was certain to be the best and 0 when a treatment was certain to be the worst. If a treatment always ranks first, then it will have 100% SUCRA, and if it always ranks last, it will have 0 SUCRA. This enabled us to rank the treatments overall. For example, treatment NE/DB emerged as the best, followed by TP, NE/EP, and last came DB

**Fig. 5:**
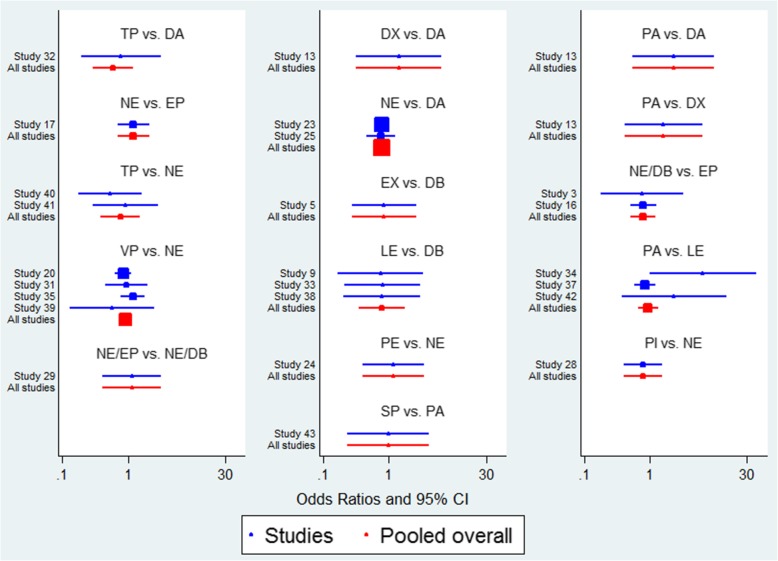
Forest plot in direct comparisons for evaluation of 28-day mortality. A blue line represents a single RCT study comparing two vasoactive medications, and a red line synthesizes multiple of studies that compared these two medications. Line length indicates the confidence interval of the results. A shorter line corresponds to larger sample size and potentially more reliable results. Solid box quantifies the relative contribution of the specific study to the overall meta-analysis, where larger box corresponds to larger contributions. Number 1 on the *x*-axis is the null value for OR = 1. The site of the spot to the left or right of 1 on the horizontal axis represents the favorable tendency to reduce 28-day mortality. The study which the ID number in the figure corresponds to is shown in Table 1

**Fig. 6:**
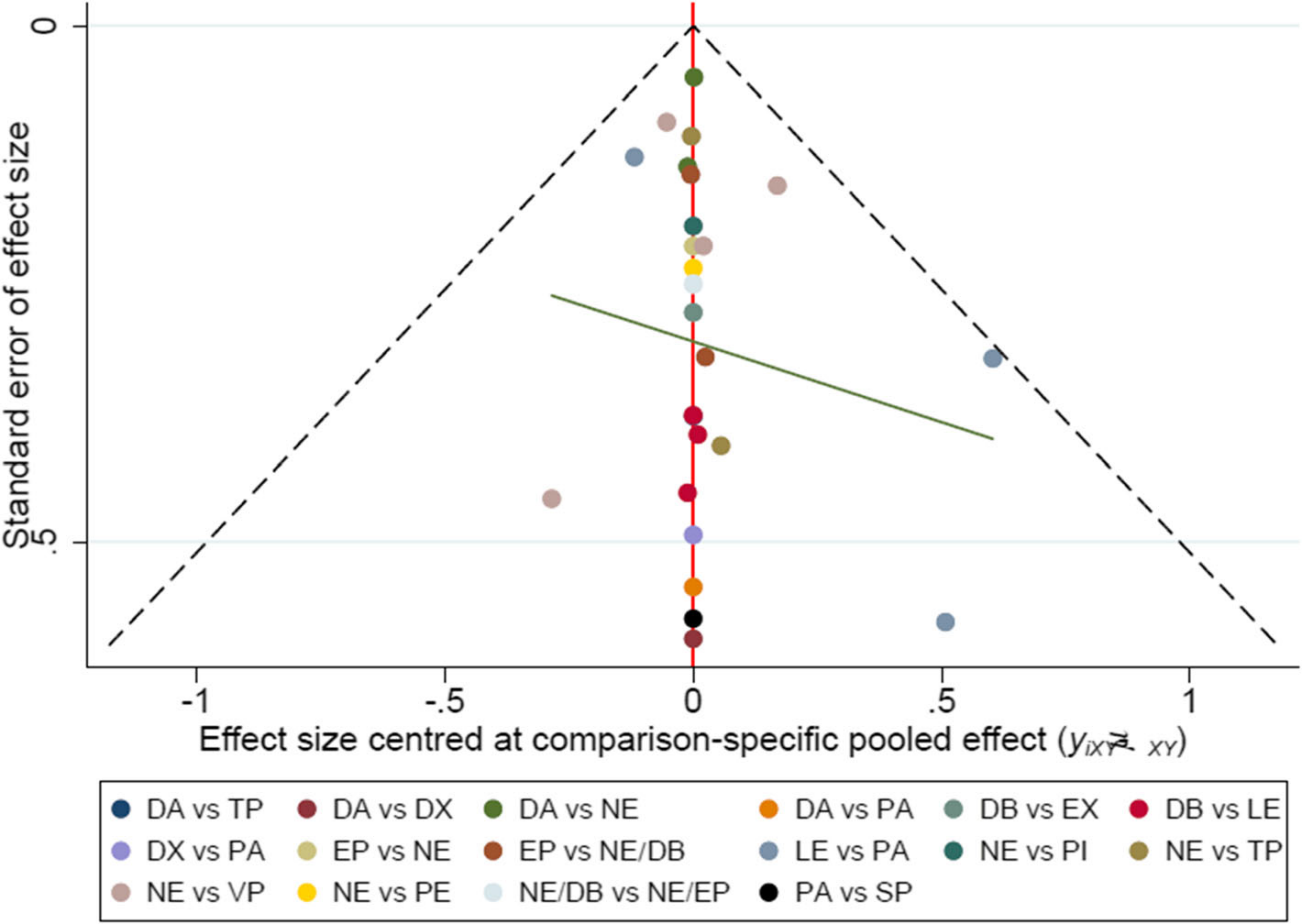
Funnel plot for 28-day mortality, with a complex evidence network including 16 sets of head-to-head randomized trials: treatment DA versus TP, DA versus DX, DA versus NE, DA versus PA, DB versus EX, DB versus LE, DX versus PA, EP versus NE, EP versus NE/DB, LE versus PA, NE versus PI, NE versus TP, NE versus VP, NE versus PE, NE/DB versus NE/EP, and PA versus SP. Single markers represented the individual primary studies, while the dashed vertical line showed the summary effect estimate, and the dashed oblique lines showed the 95% confidence intervals at varying degree of precision

A total of 24 trials with 5150 patients, which were compared to 15 treatments, were included in this analysis. There was no heterogeneity among the trials reporting 28-day mortality (*χ*^2^ = 12.46, *P* = 0.96, *I*^2^ = 0). The network geometry was shown in Fig. 3. Norepinephrine was used most frequently to assess 28-day mortality. The direct comparison between norepinephrine and dopamine was most frequent. Treatments ranking based on SUCRA values, which were shown in Fig. 4, from largest to smallest, were as follows: NE/DB 85.9%, TP 75.1%, NE/EP 74.6%, PI 74.1%, EP 72.5%, VP 66.1%, NE 59.8%, PE 53.0%, DA 42.1%, DX 38.2%, SP 27.0%, PA 24.3%, EX 22.8%, LE 21.5%, and DB 13.3%. The forest plot for direct comparison was shown in Fig. 5. The funnel plot for 28-day mortality was presented in Fig. 6. The evidence of publication bias for RR of 28-day mortality was not detected. The contribution plot, inconsistency analysis, and forest plot for all comparisons were presented in Additional file 1.

#### ICU mortality

A total of 18 trials with 1466 patients, which were compared to 11 treatments, were included in this analysis. No heterogeneity was found among trials that reported ICU mortality (*χ*^2^ = 13.75, *P* = 0.68, *I*^2^ = 0%). Similarly, norepinephrine was used most frequently to assess ICU mortality. The direct comparison between norepinephrine and vasopressin was the most frequent. Treatments ranking based on SUCRA values, from largest to smallest, were as follows: TP/NE 86.4%, TP 80.3%, TP/DB/NE 65.7%, VP/NE 62.8%, NE 57.4%, VP 56.5%, PE 48.4%, DA 33.0%, PA 27.5%, LE 22.1%, and DB 9.9%.

### Secondary outcomes

#### ICU length of stay

A total of 12 trials with 3541 patients, which were compared to 7 interventions, were included in this analysis. Significant heterogeneity was found among trials reporting ICU length of stay (*χ*^2^ = 98.73, *P* < 0.01, *I*^2^ = 89%). After omitting 1 trial (De Backer et al. 2010), the *I*^2^ value was reduced to 66%. Norepinephrine was most frequently used to assess ICU LOS. The direct comparison between norepinephrine and dopamine was most frequent. Treatments ranking based on SUCRA values, from largest to smallest, were as follows: TP 76.9%, DA 68.1%, PE 59.7%, NE 49.1%, TP/DB/NE 41.7%, TP/NE 33.7%, and VP 20.9%.

#### Hospital LOS

A total of 7 trials with 3003 patients, which were compared to 4 interventions, were included in this analysis. There was a significant heterogeneity among trials for reporting hospital LOS (*χ*^2^ = 17.36, *P* = 0.008, *I*^2^ = 65%). After omitting 1 study (Mehta S et al. 2013), no heterogeneity was found around these trials (*χ*^2^ = 2.19, *P* = 0.82, *I*^2^ = 0%). Norepinephrine was most frequently used to assess hospital LOS. The direct comparison between norepinephrine and dopamine was most frequent. Treatments ranking based on SUCRA values, from largest to smallest, were as follows: VP 82.7% (95%CI, 79.27 to 83.48), DA 49.8%, TP 46.2%, and NE 21.3%.

### Adverse events

#### Myocardial infarction

A total of 7 trials with 3793 patients, which were compared to 8 treatments, reported the incidence of myocardial infarction. The most common incidence was reported with NE/EP 3.33% (*n* = 1 of 30), followed by EP 3.11% (*n* = 5 of 161), and then VP 3.10% (*n* = 19 of 613), NE 3.03% (*n* = 43 of 1417), DA 2.21% (*n* = 19 of 858), NE/DB 2.01% (*n* = 4 of 199), LE 1.16% (*n* = 3 of 258), and PA 0.39% (*n* = 1 of 257).

#### Arrhythmia

A total of 7 trials with 4022 patients, which were compared to 8 treatments, reported the incidence of arrhythmia. The most common incidence was reported with DA 26.01% (*n* = 258 of 992), followed by EP 22.98% (*n* = 37 of 161), and then NE/DB 20.60% (*n* = 41 of 199), NE/EP 20.0% (*n* = 6 of 30), NE 8.33% (*n* = 127 of 1525), LE 5.81% (*n* = 15 of 258), PA 2.33% (*n* = 6 of 257), and VP 1.67% (*n* = 10 of 600).

#### Peripheral ischemia

The limb ischemia, skin ischemia, mesenteric ischemia, and digital ischemia contributed to peripheral ischemia. A total of 5 trials with 3255 patients, which were compared to 6 treatments, reported the incidence of peripheral ischemia. The most common incidence was reported with NE/EP 10.0% (*n* = 3 of 30), followed by DA 6.53% (*n* = 56 of 858), and then NE/DB 4.02% (*n* = 8 of 199), VP 4.0% (*n* = 24 of 600), NE 3.13% (*n* = 44 of 1407), and EP 1.24% (*n* = 2 of 161).

### GRADE evaluation

As shown in Table 2, GRADE evaluations were conducted for 28-day mortality. The other outcomes of GRADE evaluations probably had equivalent or worse quality.

**Table 2:** GRADE evaluation for 28-day mortality

Comparisons	Number of comparisons	Contribution to the network (%)	Confidence
DA vs. DX	1	4.0	High
DA vs. NE	2	4.3	High
DA vs. PA	1	2.4	High
DA vs. TP	1	10.1	Moderate
DB vs. EX	1	0.7	High
DB vs. LE	3	0.7	Moderate
DX vs. PA	1	5.1	High
NE vs. EP	1	17.9	High
EP vs. NE/DB	2	11.4	Moderate
LE vs. PA	3	5.3	High
NE/DB vs. NE/EP	1	7.3	High
NE vs. PE	1	4.2	Low
NE vs. PI	1	6.6	Moderate
NE vs. TP	2	9.1	Low
NE vs. VP	4	6.8	High
SP vs. PA	1	4.1	High

The contribution represents the proportion of each comparison in the whole network meta-analysis. Higher proportion indicates more contribution to the NMA. GRADE classifies the overall quality of a body of evidence for each outcome across studies into four levels: high, moderate, low, and very low. The rating reflects the extent of the confidence in the estimates of intervention’s effects

## Discussion

In this multiple-treatments meta-analysis of randomized controlled trials for septic shock, a comprehensive literature search was performed with no restriction for publication date, to ensure maximum coverage of existing trials. This multiple-treatments meta-analysis showed that the administration of norepinephrine combined with dobutamine may be associated with lower 28-day mortality among septic shock patients compared to other vasoactive medications. In addition, norepinephrine plus terlipressin was associated with decreased ICU mortality compared to other medications. Furthermore, terlipressin and vasopressin were associated with reduced length of stay in ICU and hospital, respectively.

There were eight systematic reviews and meta-analyses that evaluated the effects of vasoactive medications among patients with septic shock [9, 10, 22–27]. This multiple-treatments meta-analysis is the first one that altogether considered mortality, length of stay, and adverse events as outcomes. Findings in this study with those studies were compared in Table 3. There were four pairwise meta-analyses that compared the efficacy between two interventions [9, 10, 22, 23] and four network meta-analyses that compared the efficacy among multiple interventions [24–27]. Different from the existing studies, our research had some remarkable characteristics. Firstly, the previous conventional meta-analysis usually focused on pairwise comparisons of two therapeutic measures, but this study is a network meta-analysis, which was able to construct two or more interventions into a network structure, enabling computation of relative effectiveness from both direct one-to-one and indirect comparison with multiple interventions that were not evaluated in a direct assessment. Secondly, all the published NMA studies did not use the SUCRA values to determine the hierarchy of interventions for outcomes. It is the first time that the effectiveness of these vasoactive medications was ranked objectively according to their SUCRA values. The SUCRA values showed the percentage of effectiveness for each intervention compared to a hypothetical optimal intervention, which was considered the best without uncertainty [18, 28–30]. Consequently, NMA was able to provide the highest level of evidence for clinical guidelines. Thirdly, previous studies did not include the latest RCTs, due to the time of publication and sample sizes were not large enough for accurate assessment of these treatments. Missing studies and an inadequate number of patients could substantially impact the outcome of NMA. In this NMA, a comprehensive assessment of the various therapeutic interventions with higher precision and larger sample sizes was performed.

**Table 3:** Comparison of our study with relevant studies

Author	Publication date	Type	Number of studies	Number of included patients	Number of inventions	SUCRA	Survive benefit
This study		NMA	43	5767	17	Yes	NE/DB
De Backer D	2012	MA	6	1408	2	–	NE
Vasu TS	2012	MA	6	2043	2	–	NE
Serpa Neto A	2012	MA	9	998	2	–	VP
Avni T	2015	NMA	32	3544	5	No	NE
Zhou F	2015	NMA	21	3819	11	No	NE
Nagendran M	2016	NMA	13	3146	9	No	VP
Wang B	2017	MA	10	816	4	–	None
Belletti A	2017	NMA	33	3470	16	No	Inodilators

This multiple-treatments meta-analysis included a total of 17 different vasoactive interventions, which were used frequently by clinicians to treat patients with septic shock for improving their hemodynamic status. Bayesian NMA was used to estimate the comparative efficacy of the combined use of vasoactive agents, aiming to identify the most preferable regimen to improve blood pressure and heart function in the clinical setting. Meanwhile, more evidence-based information on selection of the most optimal treatment for septic shock were attempted to present. The SUCRA values were used to identify which vasoactive medications were associated with lower mortality and hospital stay among patients with septic shock. For an overall assessment of the best therapeutic option, a cumulative SUCRA score was derived by summing the individual SUCRA values for each endpoint for all 17 interventions. Taking all 4 key clinical endpoints into consideration, the 3 best options in the order of final ranking were as follows: norepinephrine plus dobutamine, norepinephrine plus epinephrine, and terlipressin.

Norepinephrine has been recognized as the first-line treatment for achieving hemodynamic goals for septic shock in international guidelines [1]. When the administration of norepinephrine alone was not able to improve the hemodynamic status, other vasoactive medications would be added. According to the results of this network meta-analysis, dobutamine is superior to other vasoactive medications in reducing 28-day mortality when combined with norepinephrine. Nevertheless, there is a potential risk when giving dobutamine to septic shock patients with normal cardiac function. As one of the inotropic medications, dobutamine may cause atrial fibrillation, multifocal atrial tachycardia, ventricular tachycardia, and fibrillation. Administration of vasopressor plus inotrope with high β-adrenergic component may contribute to a worse outcome and increase the incidence of arrhythmias [31]. It is essential to therefore identify patients who may benefit from dobutamine. Patients with a low cardiac output due to impaired contractility may probably benefit from dobutamine. For these patients, it is an option to add dobutamine when norepinephrine alone cannot improve the hemodynamic status. The dose of dobutamine should be adjusted according to the patients’ response. If adverse events occur, dobutamine should be discontinued immediately.

This study still had some limitations. Firstly, by the nature of meta-analysis in general, the results of this paper were dependent on the quality of available studies. Some included studies were small-scale single-centered trials. The results and conclusions should therefore be interpreted with caution, because of relatively small sample sizes. Secondly, some included studies did not really randomized patients to receive the vasoactive medications in a sustained manner. This was a potential factor that may have influenced the results of this network meta-analysis. Thirdly, four studies were included to compare the effect on mortality between norepinephrine plus dobutamine and other vasoactive medications. Majority of these studies did not use dobutamine according to the change of cardiac function, which probably varied the results. Fourthly, NMA included both direct and indirect comparisons, which contributed to the reduced statistical power and uncertainty on ranking results. More trials are thus expected to help accurately assess the clinical value for the combined use of vasoactive medications. Especially, direct comparisons between norepinephrine plus dobutamine and norepinephrine alone are needed to further confirm the conclusions and justify the combination of vasoactive medications for patients with septic shock. Fifthly, in some studies, more than one vasoactive medication was applied in both treatment and control groups during the trial, and the combination of different kinds of vasoactive medications without the same standard therapeutic drug-based protocol might complicate the analyses of the results.

## Conclusions

The results from this network meta-analysis suggest that the use of norepinephrine plus dobutamine was associated with lower 28-day mortality for septic shock, especially among patients with low cardiac output.

## Additional file

Additional file 1: **Table S1.** Study characteristics: management description of included randomized controlled trials. **Table S2.** Assessment of risk of bias. **Figure S1-S3.** Forest plot for the overall comparisons, contribution plot, and inconsistency analysis for 28-day mortality. **Figure S4-S9.** Network geometry, SUCRA, forest plot, contribution plot, inconsistency analysis, and funnel plot for ICU mortality. **Figure S10-S15. **Network geometry, SUCRA, forest plot, contribution plot, inconsistency analysis, and funnel plot for ICU length of stay. **Figure S16-S21.** Network geometry, SUCRA, forest plot, contribution plot, inconsistency analysis, and funnel plot for hospital length of stay. (PDF 1411 kb)

## Abbreviations

CENTRAL: Cochrane Central Register for Controlled Trials
CI: Confidence interval
DA: Dopamine
DB: Dobutamine
DX: Dopexamine
EP: Epinephrine
EX: Enoximone
GRADE: Grades of Recommendation, Assessment, Development and Evaluation
ICU: Intensive care unit
LE: Levosimendan
LOS: Length of stay
MAP: Mean arterial pressure
NE: Norepinephrine
NMA: Network meta-analysis
PA: Placebo
PE: Phenylephrine
PI: Pituitrin
RCTs: Randomized controlled trial studies
RR: Risk ratio
SMD: Standardized mean difference
SP: Selepressin
SUCRA: Surface under the cumulative ranking curve
TP: Terlipressin
VP: Vasopressin
