# Supplementary material for: Comparative efficacy of vasoactive medications in patients with septic shock: a network meta-analysis of randomized controlled trials
Source: Crit Care. 2019 May 14;23:168. doi: 10.1186/s13054-019-2427-4 (PMC6518735; doi:10.1186/s13054-019-2427-4)

SUPPLEMENTARY APPENDIX

Comparative efficacy of vasoactive medications in patients with septic shock: a network meta-analysis of randomized controlled trials.

Search strategy for PubMed	page 2
Included Studies	page 4
Table S1	page 11
Table S2	page 38
Figure S1-S3	page 42
Figure S4-S9	page 45
Figure S10-S15	page 51
Figure S16-S21	page 57

Search strategy for Medline

1. sepsis; ti, ab.
2. Septic shock; ti, ab.
3. 1 or 2
4. vasoactive agent; ti, ab.
5. norepinephrine; ti, ab.
6. epinephrine; ti, ab.
7. adrenaline; ti, ab.
8. dopamine; ti, ab.
9. dobutamine; ti, ab.
10. phenylephrine; ti, ab.
11. vasopressin; ti, ab.
12. terlipressin； ti,ab.
13. Angiotensin; ti,ab.
14. 4 or 5 or 6 or 7 or 8 or 9 or 10 or 11 or 12 or 13
15. randomized controlled trial; pt.
16. controlled clinical trial; pt.
17. randomized; ti, ab.
18. placebo; ti, ab.
19. randomly; ti, ab.
20. trial; ti.
21. 15 or 16 or 17 or 18 or 19 or 20

22. 3 and 14 and 21

Included studies

1. Martin C, Papazian L, Perrin G, Saux P, Gouin F. Norepinephrine or dopamine for the treatment of hyperdynamic septic shock? Chest. 1993, 103(6): 1826-31.
2. Marik PE, Mohedin M. The contrasting effects of dopamine and norepinephrine on systemic and splanchnic oxygen utilization in hyperdynamic sepsis. JAMA. 1994, 272(17):1354-7.
3. Levy B, Bollaert PE, Charpentier C, Nace L, Audibert G, Bauer P, et al. Comparison of norepinephrine and dobutamine to epinephrine for hemodynamics, lactate metabolism, and gastric tonometric variables in septic shock: a prospective, randomized study. Intensive Care Med. 1997, 23(3):282-7.
4. Malay MB, Ashton RC Jr, Landry DW, Townsend RN. Low-dose vasopressin in the treatment of vasodilatory septic shock. J Trauma. 1999, 47(4):699-703; discussion 703-5.
5. Kern H, Schröder T, Kaulfuss M, Martin M, Kox WJ, Spies CD. Enoximone in contrast to dobutamine improves hepatosplanchnic function in fluid-optimized septic shock patients. Crit Care Med. 2001, 29(8):1519-25.
6. Seguin P, Bellissant E, Le Tulzo Y, Laviolle B, Lessard Y, Thomas R, et al. Effects of epinephrine compared with the combination of dobutamine and norepinephrine on gastric perfusion in septic shock. Clin Pharmacol Ther. 2002, 71(5):381-8.
7. Patel BM, Chittock DR, Russell JA, Walley KR. Beneficial effects of short-term vasopressin infusion during severe septic shock. Anesthesiology. 2002, 96(3):576-82.

8. Dünser MW, Mayr AJ, Ulmer H, Knotzer H, Sumann G, Pajk W, et al. Arginine vasopressin in advanced vasodilatory shock: a prospective, randomized, controlled study. Circulation. 2003, 107(18):2313-9.
9. Morelli A, De Castro S, Teboul JL, Singer M, Rocco M, Conti G, et al. Effects of levosimendan on systemic and regional hemodynamics in septic myocardial depression. Intensive Care Med. 2005, 31(5):638-44.
10. Albanèse J, Leone M, Delmas A, Martin C. Terlipressin or norepinephrine in hyperdynamic septic shock: a prospective, randomized study. Crit Care Med. 2005, 33(9):1897-902.
11. Luckner G1, Dünser MW, Stadlbauer KH, Mayr VD, Jochberger S, Wenzel V, et al. Cutaneous vascular reactivity and flow motion response to vasopressin in advanced vasodilatory shock and severe postoperative multiple organ dysfunction syndrome. Crit Care. 2006, 10(2):R40.
12. Seguin P, Laviolle B, Guinet P, Morel I, Mallédant Y, Bellissant E. Dopexamine and norepinephrine versus epinephrine on gastric perfusion in patients with septic shock: a randomized study [NCT00134212]. Crit Care. 2006, 10(1):R32.
13. Schmoelz M, Schelling G, Dunker M, Irlbeck M. Comparison of systemic and renal effects of dopexamine and dopamine in norepinephrine-treated septic shock. J Cardiothorac Vasc Anesth. 2006, 20(2):173-8.
14. Lauzier F, Lévy B, Lamarre P, Lesur O. Vasopressin or norepinephrine in early hyperdynamic septic shock: a randomized clinical trial. Intensive Care Med. 2006, 32(11):1782-9.

15. Mathur SK, Dhunna R, Chakraborty A. Comparison of norepinephrine and dopamine in the management of septic shock using impedance cardiography. Indian J Crit Care Med. 2007, 11(4):184-191.

16. Annane D, Vignon P, Renault A, Bollaert PE, Charpentier C, Martin C, et al. Norepinephrine plus dobutamine versus epinephrine alone for management of septic shock: a randomised trial. Lancet. 2007, 370(9588):676-84.

17. Myburgh JA, Higgins A, Jovanovska A, Lipman J, Ramakrishnan N, Santamaria J, et al. A comparison of epinephrine and norepinephrine in critically ill patients. Intensive Care Med. 2008, 34(12):2226-34.

18. Morelli A, Ertmer C, Lange M, Dünser M, Rehberg S, Van Aken H, et al. Effects of short-term simultaneous infusion of dobutamine and terlipressin in patients with septic shock: the DOBUPRESS study. Br J Anaesth. 2008, 100(4):494-503.

19. Morelli A, Ertmer C, Rehberg S, Lange M, Orecchioni A, Laderchi A, et al. Phenylephrine versus norepinephrine for initial hemodynamic support of patients with septic shock: a randomized, controlled trial. Crit Care. 2008, 12(6):R143.

20. Russell JA, Walley KR, Singer J, Gordon AC, Hébert PC, Cooper DJ, et al. Vasopressin versus norepinephrine infusion in patients with septic shock. N Engl J Med. 2008, 358(9):877-87.

21. Morelli A, Ertmer C, Rehberg S, Lange M, Orecchioni A, Cecchini V, et al. Continuous terlipressin versus vasopressin infusion in septic shock (TERLIVAP): a randomized, controlled pilot study. Crit Care. 2009, 13(4):R130.

22. Alhashemi JA, Alotaibi QA, Abdullah GM, Shalabi SA. Levosimendan vs

dobutamine in septic shock. J Crit Care. 2009, 24(3):e14-5.

23. De Backer D, Biston P, Devriendt J, Madl C, Chochrad D, Aldecoa C, et al. Comparison of dopamine and norepinephrine in the treatment of shock. N Engl J Med. 2010, 362(9):779-89.
24. Jain G, Singh DK. Comparison of phenylephrine and norepinephrine in the management of dopamine-resistantseptic shock. Indian J Crit Care Med. 2010, 14(1):29-34.
25. Patel GP, Grahe JS, Sperry M, Singla S, Elpern E, Lateef O, et al. Efficacy and safety of dopamine versus norepinephrine in the management of septic shock. Shock. 2010, 33(4):375-80.
26. Morelli A, Donati A, Ertmer C, Rehberg S, Lange M, Orecchioni A, et al. Levosimendan for resuscitating the microcirculation in patients with septic shock: a randomized controlled study. Crit Care. 2010, 14(6):R232.
27. Morelli A, Donati A, Ertmer C, Rehberg S, Kampmeier T, Orecchioni A, et al. Effects of vasopressinergic receptor agonists on sublingual microcirculation in norepinephrine-dependent septic shock. Crit Care. 2011, 15(5):R217.
28. Han XD, Sun H, Huang XY, Zhang SY, Wang YD, Ren K, et al. A clinical study of pituitrin versus norepinephrine in the treatment of patients with septic shock. Zhongguo Wei Zhong Bing Ji Jiu Yi Xue. 2012, 24(1):33-7.
29. Mahmoud KM, Ammar AS. Norepinephrine supplemented with dobutamine or epinephrine for the cardiovascular support of patients with septic shock. Indian J Crit Care Med. 2012, 16(2):75-80.

30. Memiş D, Inal MT, Sut N. The effects of levosimendan vs dobutamine added to dopamine on liver functions assessed with noninvasive liver function monitoring in patients with septic shock. J Crit Care. 2012, 27(3):318.e1-6.
31. Mehta S, Granton J, Gordon AC, Cook DJ, Lapinsky S, Newton G, et al. Cardiac ischemia in patients with septic shock randomized to vasopressin or norepinephrine. Crit Care. 2013, 17(3):R117.
32. Hua F, Wang X, Zhu L. Terlipressin decreases vascular endothelial growth factor expression and improves oxygenation in patients with acute respiratory distress syndrome and shock. J Emerg Med. 2013, 44(2):434-9.
33. Fang M, Dong S. Effects of levosimendan on hemodynamics and cardiac function in patients with septic shock. Zhonghua Wei Zhong Bing Ji Jiu Yi Xue. 2014, 26(10):692-6.
34. Torraco A, Carrozzo R, Piemonte F, Pastore A, Tozzi G, Verrigni D, et al. Effects of levosimendan on mitochondrial function in patients with septic shock: a randomized trial. Biochimie. 2014,102:166-73.
35. Gordon AC, Mason AJ, Thirunavukkarasu N, Perkins GD, Cecconi M, Cepkova M, et al. Effect of Early Vasopressin vs Norepinephrine on Kidney Failure in Patients With Septic Shock: The VANISH Randomized Clinical Trial. JAMA. 2016, 316(5):509-18.
36. Xiao X, Zhang J, Wang Y, Zhou J, Zhu Y, Jiang D, et al. Effects of terlipressin on patients with sepsis via improving tissue blood flow. J Surg Res. 2016, 200(1):274-82.

37. Gordon AC, Perkins GD, Singer M, McAuley DF, Orme RM, Santhakumaran S, et al. Levosimendan for the Prevention of Acute Organ Dysfunction in Sepsis. N Engl J Med. 2016, 375(17):1638-1648.
38. Meng JB, Hu MH, Lai ZZ, Ji CL, Xu XJ, Zhang G, et al. Levosimendan Versus Dobutamine in Myocardial Injury Patients with Septic Shock: A Randomized Controlled Trial. Med Sci Monit. 2016, 22:1486-96.
39. Barzegar E, Ahmadi A, Mousavi S, Nouri M, Mojtahedzadeh M. The Therapeutic Role of Vasopressin on Improving Lactate Clearance During and After Vasogenic Shock: Microcirculation, Is It The Black Box? Acta Med Iran. 2016, 54(1):15-23.
40. Choudhury A, Kedarisetty CK, Vashishtha C, Saini D, Kumar S, Maiwall R, et al. A randomized trial comparing terlipressin and noradrenaline in patients with cirrhosis and septic shock. Liver Int. 2017, 37(4):552-561.
41. Chen Z, Zhou P, Lu Y, Yang C. Comparison of effect of norepinephrine and terlipressin on patients with ARDS combined with septic shock: a prospective single-blind randomized controlled trial. Zhonghua Wei Zhong Bing Ji Jiu Yi Xue. 2017, 29(2):111-116.
42. Hajjej Z, Meddeb B, Sellami W, Labbene I, Morelli A, Ferjani M. Effects of Levosimendan on Cellular Metabolic Alterations in Patients With Septic Shock: A Randomized Controlled Pilot Study. Shock. 2017, 48(3):307-312.
43. Russell JA, Vincent JL, Kjølbye AL, Olsson H, Blemings A, Spapen H, et al. Selepressin, a novel selective vasopressin V1A agonist, is an effective substitute for norepinephrine in a phase IIa randomized, placebo-controlled trial in septic shock

patients. Crit Care. 2017, 21(1):213.

Table S1 Studies characteristics: management description of included randomized controlled trials

ID	Source	Group	Management description	Baseline characteristics								
				APACHE II	SOFA	MAP	HR	CI	DO_2	VO_2	PH	Lactate
1	Martin C 1993	A	Dopamine: started at a dose of 2.5µg/kg/min, to a maximum dose of 25µg/kg/min. If hemodynamic and metabolic abnormalities were not corrected with the maximum dose of one drug, the other was added.	30± 1.2	NA	53±8	110 ±20	5.4± 1.1	745±2 06	178 ±65	NA	4.8±3.2
		B	Norepinephrine: started at a dose of 0.5µg/kg/min, to a maximum dose of 5µg/kg/min. If hemodynamic and metabolic abnormalities were not corrected with the maximum dose of one drug, the other was added.	31±1.3	NA	54±10	113 ±21	5.3± 1.3	801±2 31	208 ±70	NA	4.8± 1.6
2	Marik PE 1994	A	Norepinephrine: titrated during a period of 20 minutes to achieve a MAP greater than 75 mm Hg.	18±1	NA	65±4	105 ±5	4.2± 0.5	498±7 7	145 ±21	NA	1.8±0.5
		B	Dopamine: titrated during a period	17±2	NA	63±2	121	4.2±	573±5	183	NA	2.2±0.4

			of 20 minutes to achieve a MAP greater than 75 mm Hg, and to keep the pulse rate less than 150 beats per minute.				±4	0.4	4	±29		
3	Levy B 1997	A	Epinephrine: Patients with a CI> 3.5 l/min/m^2 and a MAP≤ 60 mmHg after dopamine 20 mg/kg/min started epinephrine infusion with a dose of 0.3 mg/kg/min. The infusion rate of epinephrine was titrated on MAP at 5-min intervals to obtain a MAP > 80 mmHg with a stable or increased CI.	23±4.6	NA	60±8	121 ±19	4.1± 1	NA	NA	7.35 ±0.0 7	3.1±1.5
		B	Norepinephrine/Dobutamine: Patients with a CI> 3.5 l/min/m^2 and a MAP≤ 60 mmHg after dopamine 20 mg/kg/min started norepinephrine infusion with a dose of 0.3 mg/kg/min and dobutamine was infused at a fixed dose of 5 mg/kg/min. The infusion rate of	24±5.8	NA	60±8	125 ±15	4.0± 1	NA	NA	7.37 ±0.1 1	3.1±1.5

			norepinephrine was titrated on MAP at 5-min intervals to obtain a MAP > 80 mmHg with a stable or increased CI.									
4	Malay MB 1999	A	Placebo: normal saline was infused.	26±3	NA	66.0±6.0	116.0±7.0	5.6±0.9	709.0±71.0	NA	NA	NA
		B	Vasopressin: started at a dose of 0.04U/min.	27±2	NA	64.0±6.0	126.0±13.0	4.3±0.5	593.0±63.0	NA	NA	NA
5	Kern H 2001	A	Enoximone: 2.5 mg/kg/min initially, increased by 2.5 mg/kg/min every 2 hours, up to a maximum of 10 mg/kg/min).	15 (11,21)	NA	81 (76,86)	103 (95,111)	4.9 (3.9,6.2)	721 (524,921)	175 (132,199)	NA	1.6 (1.2,3.5)
		B	Dobutamine: 5 mg/kg/min initially, increased by 5 mg/kg/min every 2 hours, up to a maximum of 20 mg/kg/min.	19 (11,22)	NA	84 (77,88)	103 (86,118)	4.8 (3.4,5.8)	687 (508,858)	158 (140,183)	NA	1.8 (1.3,3.1)
6	Seguin P 2002	A	Epinephrine: titrated from 0.1 µg/kg/min, with 0.2 µg/kg/min increases every 5 minutes.	NA	10 ± 3	54 ± 8	97 ± 17	3.2 ± 1.1	NA	NA	NA	3.9 ± 2.6
		B	Dobutamine/ Norepinephrine:	NA	10 ± 3	51 ± 8	99 ±	3.0	NA	NA	NA	5.3 ±

			norepinephrine titrated from 0.1 μg/kg/min, with 0.2 μg/kg/min increases every 5 minutes, while dobutamine was continuously infused at 5 μg/kg/min.				26	± 1.3				4.3
7	Patel BM 2002	A	Norepinephrine: During the initial 60 min of the pre- 4h infusion protocol, norepinephrine was titrated (infusion increased by 7 ml/h every 5–10 min), and the pre-study vasopressor agent was titrated down to maintain mean arterial pressure constant at a level determined by the attending intensive care physician. Norepinephrine titrated at a dose of 2 μg/min, to a maximum dose of 16 μg/min.	24(19,30)	NA	68(65, 70)	97(89,110)	5.0(3.8, 5.6)	NA	NA	NA	NA
		B	Vasopressin: During the initial 60 min of the pre- 4h infusion protocol, norepinephrine was titrated (infusion increased by 7 ml/h every	22(20,27)	NA	69(65, 72)	102(90,110)	4.8(3.5, 5.5)	NA	NA	NA	NA

			5–10 min), and the pre-study vasopressor agent was titrated down to maintain mean arterial pressure constant at a level determined by the attending intensive care physician. Vasopressin titrated at a dose of 0.01 u/min, to a maximum dose of 0.08 u/min.									
8	Dünser MW 2003	A	Arginine vasopressin (AVP): additional AVP infusion was administered, including infusion of AVP at a constant rate of 4 U/h. No bolus injections were given. NE infusion was adjusted to maintain MAP ≥70 mm Hg. When NE requirements decreased to ＜0.3 μg/kg/min. AVP infusion was tapered off stepwise according to the response of MAP to AVP reductions.	NA	NA	63±7	115 ±17	NA	NA	NA	7.35 ±0.11	4.8±4.4
		B	Norepinephrine: MAP ≥70 mm Hg was achieved by adjusting NE	NA	NA	67±8	103 ±20	NA	NA	NA	7.34 ±0.1	4.5±4.7

			infusion as necessary. For those patients in whom NE requirements exceeded 2.26 μg/kg/min, additional AVP infusion was initiated at 4 U/h.								1	
9	Morelli A 2005	A	Dobutamine: patients received norepinephrine to maintain MAP between 70 and 80 mmHg. Dobutamine 5μg/kg/min was added to norepinephrine infusion. This therapeutic regimen was maintained during the first 48 h of the study. The dobutamine infusion was then stopped for a short time necessary to start the syringe-pumps. Then patients received an infusion of dobutamine (5μg/kg/min).	23.7±2.1	NA	74.7± 2.4	115 ±7.3	4.2± 0.3	721±5 9	225 ±23	NA	5.2±1.1
		B	Levosimendan: patients received norepinephrine to maintain MAP between 70 and 80 mmHg. Dobutamine 5μg/kg/min was added to norepinephrine infusion. This	24.4±1.6	NA	76.2± 2.8	114 ±8.3	4.1± 0.2	715±5 8	223 ±36	NA	4.9±1.2

			therapeutic regimen was maintained during the first 48 h of the study. The dobutamine infusion was then stopped for a short time necessary to start the syringe-pumps. Then patients received an infusion of levosimendan (0.2μg/kg/min) without an initial bolus loading dose.									
10	Albanèse J 2005	A	Norepinephrine: started at a dose of 0.3 μg/kg/min followed by 0.3 μg/kg/min increments at 4-min intervals to raise MAP to 65 to 75 mm Hg.	29 (24,31)	NA	54 (49,61)	118 (84, 135)	5.1 (4.1, 6.1)	NA	NA	7.40 (7.22,7.46)	NA
		B	Terlipressin: patients were given one bolus of 1 mg of the drug equivalent to 0.03– 0.04 UI/min of vasopressin. A second bolus of 1 mg was given if, after 20 minutes, MAP was ＜65 mm Hg.	28 (24,30)	NA	54 (48,62)	119 (811,134)	5.0 (4.0, 6.2)	NA	NA	7.40 (7.23,7.45)	NA
11	Luckner G 2006	A	Vasopressin/ Norepinephrine: supplementary AVP was infused at a	NA	NA	64±5	NA	4.1± 1.2	NA	NA	7.30 ±	5.9 ± 6.7

			continuous rate of 4 IU/hour; no bolus injections were administered. Norepinephrine infusion was adjusted to maintain MAP above 65 mmHg.								0.12	
		B	Norepinephrine: a MAP above 65 mmHg was achieved by adjusting the norepinephrine dosage.	NA	NA	64±9	NA	3.5± 0.9	NA	NA	7.33 ± 0.1	7.6 ± 6.1
12	Seguin P 2006	A	Epinephrine: titrated from 0.2 μg/kg/min with 0.2 μg/kg/min increments every 3 minutes until MAP reached 70 to 80 mmHg.	NA	10 ± 4	52 ± 7	94 ± 18	NA	NA	NA	NA	NA
		B	Dopexamine/ Norepinephrine: titrated from 0.5 μg/kg/min with 0.5 μg/kg/min increments every 3 minutes for dopexamine and from 0.2 μg/kg/min with 0.2 μg/kg/min increments every 3 minutes for norepinephrine until MAP reached 70 to 80 mmHg.	NA	10 ± 3	56 ± 8	102 ± 17	NA	NA	NA	NA	NA
13	Schmoelz	A	Dopexamine: titrated with 2	19.50±6.9	11.40	85.70	87.9	NA	NA	NA	NA	2.42±1.

	M 2006		μg/kg/min	5	±3.73	±9.88	5±2<br>3.67					31
		B	Dopamine: titrated with 3μg/kg/min	20.38±7.8<br>1	11.00<br>±3.80	82.57<br>±13.7<br>4	89.5<br>2±1<br>7.60	NA	NA	NA	NA	1.92±1.<br>53
		C	Placebo: An equivalent volume of placebo was administered.	23.40±7.2<br>1	12.25<br>±3.82	85.85<br>±13.3<br>2	89.2<br>0±2<br>4.81	NA	NA	NA	NA	2.55±2.<br>16
14	Lauzier F 2006	A	Vasopressin: titrated with 0.04–0.20 U/min, which was infused to maintain a MAP above 70 mmHg. Other vasopressive drugs were tapered and weaned as the experimental drug was increased. When maximal dosage of the experimental drug was reached (0.20 U/min), administration of the other drug (either NE or AVP) was allowed as rescue therapy if the MAP was still below 70 mmHg. Dobutamine was used if cardiac index decreased	22.8±3.4	8.5±1.<br>4	72±7	118<br>±16	4.6±<br>1.0	581±1<br>24	148<br>±30	NA	2.87±0.<br>75

			below 3 l/min/m^2 despite adequate volume resuscitation.									
		B	Norepinephrine: titrated with 0.1–2.8 µg/kg/min, which was infused to maintain a MAP above 70 mmHg. Other vasopressive drugs were tapered and weaned as the experimental drug was increased. When maximal dosage of the experimental drug was reached (2.8 µg/kg/min), administration of the other drug (either NE or AVP) was allowed as rescue therapy if the MAP was still below 70 mmHg. Dobutamine was used if cardiac index decreased below 3 l/min/m^2 despite adequate volume resuscitation.	23.5±4.2	9.3±1.4	68±10	109 ±23	4.4± 1.4	537±143	157 ±42	NA	3.33±1.75
15	Mathur SK 2007	A	Dopamine: titrated with 10-25 mcg/kg/min, with 2.5 mcg/kg/min increments every 15 minutes.	24.56±2.90	NA	NA	129.32± 8.30	5.28 ±0.53	NA	NA	NA	NA
		B	Norepinephrine: titrated with 0.5–	25.60±2.3	NA	NA	132.	5.45	NA	NA	NA	NA

			2.5 mcg/kg/min, with 0.25 mcg/kg/min increments every 15 minutes.	1			12± 7.30	±0.5 7				
16	Annane D 2007	A	Epinephrine: epinephrine started at dose of 0.2 μg/kg/min with placebo. When CI＞2.5 l/min/m², Norepinephrine or epinephrine raised by 0.2 μg/kg/min. When CI ≤2.5 l/min/m², dobutamine or placebo raised by 5μg/kg/min. The aim was to raise MAP to 70 mm Hg.	NA	11 (9,13)	70±19	NA	NA	NA	NA	NA	2.9 (1.7,5.0)
		B	Norepinephrine/Dobutamine: norepinephrine started at a dose of 0.2 μg/kg/min, with dobutamine at a dose of 5 μg/kg/min. When CI＞2.5 l/min/m², Norepinephrine or epinephrine raised by 0.2 μg/kg/min. When CI≤2.5 l/min/m², dobutamine or placebo raised by 5μg/kg/min. The aim was to raise MAP to 70 mm Hg.	NA	11 (9,14)	68±19	NA	NA	NA	NA	NA	3.3 (2.1,5.1)

17	Myburgh JA 2008	A	Epinephrine: Blinded infusions of epinephrine to achieve a MAP C70 mmHg for the duration of ICU admission.	21.8±7.4	NA	68.5±13.0	93.7±22.2	NA	NA	NA	7.3±0.1	2.7±2.4
		B	Norepinephrine: Blinded infusions of norepinephrine to achieve a MAP C70 mmHg for the duration of ICU admission.	22.2±6.9	NA	68.7±14.4	94.3±21.5	NA	NA	NA	7.3±0.3	2.5±2.3
18	Morelli A 2008	A	Norepinephrine: patients requiring norepinephrine doses of 0.9 mg/kg/min/ to maintain MAP at 70 (5) mm Hg, were randomized. Norepinephrine infusion was titrated to maintain the defined threshold MAP of 70 (5) mm Hg	NA	NA	73±4	NA	NA	NA	166±38	7.35±0.07	NA
		B	Terlipressin/ norepinephrine: patients requiring norepinephrine doses of 0.9 mg/kg/min/ to maintain MAP at 70 (5) mm Hg, were randomized. Then the patients received a single bolus dose of terlipressin 1 mg.	NA	NA	74±2	NA	NA	NA	163±42	7.37±0.06	NA

		C	Terlipressin/ Dobutamine/ Norepinephrine: patients requiring norepinephrine doses of 0.9 mg/kg/min/ to maintain MAP at 70 (5) mm Hg, were randomized. Patients was treated with a combination therapy consisting of a single bolus dose of terlipressin 1 mg and a titrated infusion of dobutamine. Dobutamine infusion was started immediately after terlipressin administration at a rate of 3 mg/kg/min and was progressively increased (in steps of 1–3 mg/kg/min). Dobutamine in doses up to 20 mg/kg/min was allowed.	NA	NA	72±3	NA	NA	NA	155 ±43	7.36 ±0.10	NA
19	Morelli A 2008	A	Phenylephrine: titrated to maintain a MAP between 65 and 75 mmHg. If the mixed-venous oxygen saturation	NA	NA	NA	NA	NA	500 ± 205	164 ± 48	7.37 ± 0.07	NA

			was <65% despite appropriate arterial oxygenation (arterial oxygen saturation ⩾ 95%) and hemoglobin concentrations ⩾ 8 g/dl, dobutamine was administered (with a maximum dose of 20 μg/kg/min) to achieve mixed-venous oxygen saturation values ⩾ 65%.									
		B	Norepinephrine: titrated to maintain a MAP between 65 and 75 mmHg. If the mixed-venous oxygen saturation was <65% despite appropriate arterial oxygenation (arterial oxygen saturation ⩾ 95%) and hemoglobin concentrations ⩾ 8 g/dl, dobutamine was administered (with a maximum dose of 20 μg/kg/min) to achieve mixed-venous oxygen saturation values ⩾ 65%.	NA	NA	NA	NA	NA	499 ± 139	173 ± 53	7.35 ± 0.09	NA

20	Russell JA 2008	A	Norepinephrine: Vasopressin (30 U) and norepinephrine (15 mg) were mixed in identical 250-ml intravenous bags of 5% dextrose in water, with final concentrations of 0.12 U of vasopressin per milliliter and 60 μg of norepinephrine per milliliter. The study-drug infusion was started at 5 ml per hour and increased by 2.5 ml per hour every 10 minutes during the first hour to achieve a constant target rate of 15 ml per hour. Thus, the blinded norepinephrine infusion was started at 5 μg per minute and titrated to a maximum of 15 μg per minute.	27.1±6.9	NA	NA	NA	NA	NA	NA	NA	NA
		B	Vasopressin: Vasopressin (30 U) and norepinephrine (15 mg) were mixed in identical 250-ml intravenous bags of 5% dextrose in water, with final concentrations	27.0±7.7	NA	NA	NA	NA	NA	NA	NA	NA

			of 0.12 U of vasopressin per milliliter and 60 µg of norepinephrine per milliliter. The study-drug infusion was started at 5 ml per hour and increased by 2.5 ml per hour every 10 minutes during the first hour to achieve a constant target rate of 15 ml per hour. Thus, the blinded vasopressin infusion was started at 0.01 U per minute and titrated to a maximum of 0.03 U per minute.									
21	Morelli A 2009	A	Terlipressin: 1.3µg/kg/h terlipressin continuous infusion plus open-label norepinephrine infusion to maintain MAP at 70±5 mm Hg.	NA	NA	53±6	95±16	4.0±1.0	NA	NA	NA	3.1±1.8
		B	Vasopressin: 0.03 U/min arginine vasopressin continuous infusion plus open-label norepinephrine infusion to maintain MAP at 70±5 mm Hg.	NA	NA	53±4	100±22	4.0±1.1	NA	NA	NA	3.0±2.4
		C	Norepinephrine: 15µg/min	NA	NA	54±3	97±	4.0±	NA	NA	NA	3.1±2.2

			norepinephrine continuous infusion plus open-label norepinephrine infusion to maintain MAP at 70±5 mm Hg.				21	1.0				
22	Alhashemi JA 2009	A	Levosimendan: study drugs were titrated incrementally to an $ScvO_2$ ≥70% with a preset maximum dose, whichever was achieved first. Patients received levosimendan 0.05 μg/kg/min intravenously, which was increased by 0.05 μg/kg/min every 30 minutes (maximum, 0.2 μg/kg/min) and was continued for 24 hours only. Hypotension (MAP＜65 mm Hg) was treated with norepinephrine infusion, titrated to a MAP 65 mm Hg or more.	26 ± 7	NA	NA	NA	NA	NA	NA	NA	NA
		B	Dobutamine: study drugs were titrated incrementally to an $ScvO_2$ ≥70% with a preset maximum dose, whichever was achieved first.	27 ± 7	NA	NA	NA	NA	NA	NA	NA	NA

			Patients received dobutamine, 5 μg/kg/min intravenously, which was increased by 5 μg/kg/min every 30 minutes (maximum, 20 μg/kg/min) and was continued for a maximum of 7 days. Hypotension (MAP＜65 mm Hg) was treated with norepinephrine infusion, titrated to a MAP 65 mm Hg or more.									
23	De Backer D 2010	A	Dopamine: Doses of dopamine could be increased or decreased by 2 μg/kg/min. If the patient was still hypotensive after the maximum dose of agent had been administered (20 μg/kg/min), open-label norepinephrine was added. If the patient was already being treated with a vasopressor at baseline, that agent was replaced as soon as possible with the trial-drug solution. If the patient was already receiving dopamine and this agent could not	20(15,28)	9(7,12)	58±13	97±27	3.11±1.35	NA	NA	7.32±0.13	2.1(1.2,4.3)

			be discontinued after introduction of the trial-drug solution, the dopamine was replaced with an open-label norepinephrine infusion. Open-label dopamine was not allowed at any time. Epinephrine and vasopressin were used only as rescue therapy. Inotropic agents could be used, if needed, to increase cardiac output.									
		B	Norepinephrine: Doses of norepinephrine could be increased or decreased by 0.02 µg/kg/min. If the patient was still hypotensive after the maximum dose of agent had been administered (0.19µg/kg/min), open-label norepinephrine was added. If the patient was already being treated with a vasopressor at baseline, that agent was replaced as soon as possible with the trial-drug solution.	20(14,27)	9(6,12)	58±13	95±25	2.77±1.16	NA	NA	7.32±0.14	2.2(1.2,3.8)

			If the patient was already receiving dopamine and this agent could not be discontinued after introduction of the trial-drug solution, the dopamine was replaced with an open-label norepinephrine infusion. Open-label dopamine was not allowed at any time. Epinephrine and vasopressin were used only as rescue therapy. Inotropic agents could be used, if needed, to increase cardiac output.									
24	Jain G 2010	A	Norepinephrine: titrated with 0.5-3.5 μg/kg/min, with 0.5 μg/kg/min increments every 30 minutes.	17.66±3.43	NA	47.55 ±4.30	151.74± 7.62	4.97 ±0.30	NA	NA	NA	3.40±0.74
		B	Phenylephrine: titrated with 0.5-8.5 μg/kg/min, with 1 μg/kg/min increments every 30 minutes.	19.11±3.11	NA	48.96 ±3.36	152.66± 7.28	5.02 ±0.56	NA	NA	NA	3.44±0.64
25	Patel GP 2010	A	Dopamine: Patients received dopamine (5-20 mcg/kg/min) as the first-line vasopressor therapy. If the predetermined maximum dose was	28±6.7	12±3.3	NA	NA	NA	NA	NA	NA	NA

			reached for the initial vasopressor, then the addition of vasopressin at a continuous infusion dose (0.04 U/min) was initiated. Patients who required additional hemodynamic support to meet the goals were then started on an infusion of phenylephrine (25-200 mcg/min), which was titrated to reach the goal hemodynamic parameters. Those patients who were found to have a low central venous oxygen saturation (ScvO2 G70%) were given dobutamine.									
		B	Norepinephrine: Patients received norepinephrine (5-20 mcg/min) as the first-line vasopressor therapy. If the predetermined maximum dose was reached for the initial vasopressor, then the addition of vasopressin at a continuous infusion dose (0.04 U/min) was initiated.	27± 6.1	12±3.2	NA	NA	NA	NA	NA	NA	NA

			Patients who required additional hemodynamic support to meet the goals were then started on an infusion of phenylephrine (25-200 mcg/min), which was titrated to reach the goal hemodynamic parameters. Those patients who were found to have a low central venous oxygen saturation ($ScvO_2$ 70%) were given dobutamine.									
26	Morelli A 2010	A	Levosimendan:	NA	NA	70 (67, 72)	96 (87, 107)	3.6 (2.9, 4.3)	431 (363,531)	111 (93, 151)	7.29 (7.25,7.34)	NA
		B	Dobutamine	NA	NA	72 (70,74)	95 (90, 106)	3.9 (2.9, 4.6)	492 (393,550)	126 (112,153)	7.28 (7.25, 7.38)	NA
27	Morelli A 2011	A	Terlipressin	NA	NA	71 (68,75)	104 (86, 113)	3.8 (3.1, 5.4)	437 (391,604)	NA	7.30 (7.24,7.	1.8 (1.2,2.9)

											34)	
		B	Arginine vasopressin	NA	NA	72 (69,75)	99 (83, 119)	4.0 (3.2, 4.9)	460 (396,677)	NA	7.31 (7.27,7.37)	2.3 (1.4,3.6)
		C	Control	NA	NA	71 (68,75)	104 (86, 111)	4.0 (3.5, 4.6)	470 (399,550)	NA	7.31 (7.28,7.36)	2.5 (1.9,3.0)
28	Han XD 2012	A	Norepinephrine	26.89±7.52	8.68±3.74	NA	NA	NA	NA	NA	NA	3.75±2.07
		B	Pituitrin	27.83±8.95	8.94±4.28	NA	NA	NA	NA	NA	NA	4.33±2.24
29	Mahmoud KM 2012	A	Norepinephrine/ Dobutamine	NA	15.2 ± 2.4	52 ± 5	102 ± 6	2.10 ± 1.1	NA	NA	7.16 ± 0.36	2.88 ± 0.49
		B	Norepinephrine/ Epinephrine	NA	14.4 ± 2.9	54 ± 4	103 ± 7	2.15 ± 1.0	NA	NA	7.17 ± 0.36	2.91 ± 0.29
30	Memis D 2012	A	Dobutamine	NA	NA	62.07 ± 1.62	NA	NA	NA	NA	NA	NA
		B	Levosimendan	NA	NA	63.07	NA	NA	NA	NA	NA	NA

						± 1.33						
31	Mehta S 2013	A	Vasopressin	28.1±8.0	NA	72.0(67.0,77.0)	NA	NA	NA	NA	7.34 (7.3, 7.4)	2.3(1.5, 3.6)
		B	Norepinephrine	29.2±7.3	NA	70.0(65.0,75.0)	NA	NA	NA	NA	7.36 (7.3, 7.4)	2.2(1.6, 4.1)
32	Hua F 2013	A	Terlipressin	19.3±9.6	NA	NA	NA	NA	NA	NA	NA	NA
		B	Dopamine	17.6±5.3	NA	NA	NA	NA	NA	NA	NA	NA
33	Fang MX 2014	A	Dobutamine	24.1±2.1	19.0± 1.4	67.6± 12.3	115.4±12.1	3.4± 0.6	NA	NA	NA	5.3±0.8
		B	Levosimendan	23.5±2.4	17.0± 1.1	68.3± 13.2	118.2±14.3	3.2± 0.6	NA	NA	NA	5.1±0.9
34	Torraco A 2014	A	Levosimendan	NA	NA	70 (70,70)	108 (95, 112)	NA	NA	NA	NA	NA
		B	Control	NA	NA	70 (67,70)	101 (93, 114)	NA	NA	NA	NA	NA
35	Gordon	A	Vasopressin	24 (19,30)	NA	71	98	NA	NA	NA	NA	2.1

	AC 2016					(62,80)	(85, 109)					(1.4,4.3)
		B	Norepinephrine	24 (20,30)	NA	68 (61,75)	99 (83, 112)	NA	NA	NA	NA	2.6 (1.4,4.5)
36	Xiao XD 2016	A	Norepinephrine	NA	NA	NA	NA	NA	NA	NA	7.32 ±0.16	3.6 ±3.2
		B	Terlipressin/Norepinephrine	NA	NA	NA	NA	NA	NA	NA	7.30 ±0.14	3.2 ±1.8
37	Gordon AC 2016	A	Levosimendan	25 (21,31)	10 (8,12)	74 (68,80)	97 (82, 111)	2.7 (2.2, 3.7)	NA	NA	NA	2.2 (1.4,3.5)
		B	Placebo	25 (21,30)	10 (7,12)	73 (67,79)	94 (80, 110)	3.3 (2.2, 4.0)	NA	NA	NA	2.3 (1.5,3.9)
38	Meng JB 2016	A	Levosimendan	18.4±4.5	4.2±1.8	67.6±2.0	116.1±7.5	3.0±0.2	716.8 ±56.2	123.2±16.9	NA	5.1±1.2
		B	Dobutamine	19.5±4.3	4.3±2.6	67.4±2.1	113.8±6.	2.9±0.3	725.5 ±58.7	125.6±1	NA	4.7±1.1

							9			3.4		
39	Barzegar E 2016	A	norepinephrine	NA	12±2.6	62.2±6.5	87.2±18	NA	NA	NA	7.32±0.07	2.0±1.1
		B	vasopressin	NA	11±3.3	65.4±6.4	90.2±19.2	NA	NA	NA	7.3±0.1	2.3±1.0
40	Choudhury A 2017	A	Terlipressin	NA	13.74±3.07	60.67±2.79	104.86±16.32	NA	NA	NA	NA	3
		B	Noradrenaline	NA	14.78±2.66	60.29±3.05	104.69±17.72	NA	NA	NA	NA	3
41	Chen Z 2017	A	norepinephrine	20.8±5.7	NA	53.8±3.6	108.5±12.6	55.18±11.84	NA	NA	NA	NA
		B	terlipressin	23.1±5.2	NA	55.1±4.1	103.5±11.5	57.01±12.50	NA	NA	NA	NA
42	Hajjej Z	A	Control	NA	NA	73	92	3.5(	452	143(	7.4(	1.7(1.4,

	2017					(62,86)	(84, 102)	3.2, 4.4)	(387,532)	87,212)	7.38,7.42)	2.2)
		B	Levosimendan	NA	NA	74 (60,84)	102 (80, 110)	4(3, 4.6)	546 (497–646)	151(71,231)	7.4(7.38,7.44)	2.1(1.7, 2.8)
43	Russell JA 2017	A	Selepressin	NA	NA	NA	NA	NA	NA	NA	NA	NA
		B	Placebo	NA	NA	NA	NA	NA	NA	NA	NA	NA

Table S2 Assessment of risk of bias

Study	Random sequence generation	Allocation concealment	Blinding of participants and personnel	Blinding of outcome assessment	Incomplete outcome data	Selective reporting	Other bias
Martin C 1993	low	low	low	low	low	low	low
Marik PE 1994	low	unclear	unclear	unclear	unclear	unclear	unclear
Levy B 1997	low	unclear	unclear	unclear	unclear	unclear	unclear
Malay MB 1999	low	low	unclear	unclear	unclear	low	low
Kern H 2001	low	low	low	low	low	low	low
Seguin P 2002	unclear	unclear	unclear	unclear	unclear	unclear	unclear
Patel BM 2002	low	low	low	low	low	low	low
Dünser MW 2003	unclear	unclear	unclear	unclear	unclear	unclear	unclear
Morelli A 2005	low	unclear	unclear	unclear	unclear	unclear	unclear
Albanèse J 2005	low	unclear	unclear	unclear	unclear	unclear	unclear
Luckner G 2006	low	high	high	high	unclear	unclear	unclear

Seguin P 2006	unclear	unclear	unclear	unclear	unclear	unclear	unclear
Schmoelz M 2006	low	low	low	low	low	low	low
Lauzier F 2006	low	low	low	low	low	low	low
Mathur SK 2007	unclear	high	high	high	unclear	unclear	unclear
Annane D 2007	low	low	low	low	low	low	low
Myburgh JA 2008	low	low	low	low	low	low	low
Morelli A 2008	low	low	unclear	unclear	low	low	low
Morelli A 2008	low	low	unclear	unclear	low	low	low
Russell JA 2008	low	low	low	low	low	low	low
Morelli A 2009	low	low	low	low	low	low	low
Alhashemi JA 2009	unclear	unclear	unclear	unclear	unclear	unclear	unclear

De Backer D 2010	low	low	low	low	low	low	low
Jain G 2010	low	high	high	low	unclear	unclear	unclear
Patel GP 2010	low	low	unclear	unclear	low	low	low
Morelli A 2010	low	low	low	low	low	low	low
Morelli A 2011	low	low	low	low	low	low	low
Han 2012	unclear	unclear	unclear	unclear	unclear	unclear	unclear
Mahmoud KM 2012	low	low	low	low	low	low	low
Memis D 2012	unclear	unclear	unclear	unclear	unclear	unclear	unclear
Mehta S 2013	low	low	low	low	low	low	low
Hua F 2013	low	low	unclear	unclear	low	low	low
Fang MX 2014	low	high	high	high	low	low	low
Torraco A 2014	low	unclear	high	high	low	low	low
Gordon AC 2016	low	low	low	low	low	low	low
Xiao XD 2016	low	unclear	high	high	low	low	low

Gordon AC 2016	low	low	low	low	low	low	low
Meng JB 2016	low	unclear	high	high	low	low	low
Barzegar E 2016	low	unclear	high	high	low	low	low
Choudhury A 2017	low	low	low	low	low	low	low
Chen Z 2017	low	high	high	high	low	low	low
Hajjej Z 2017	low	high	high	high	low	low	low
Russell JA 2017	low	low	low	low	low	low	low

Supplementary Figure S1 Forest plot for 28-day mortality

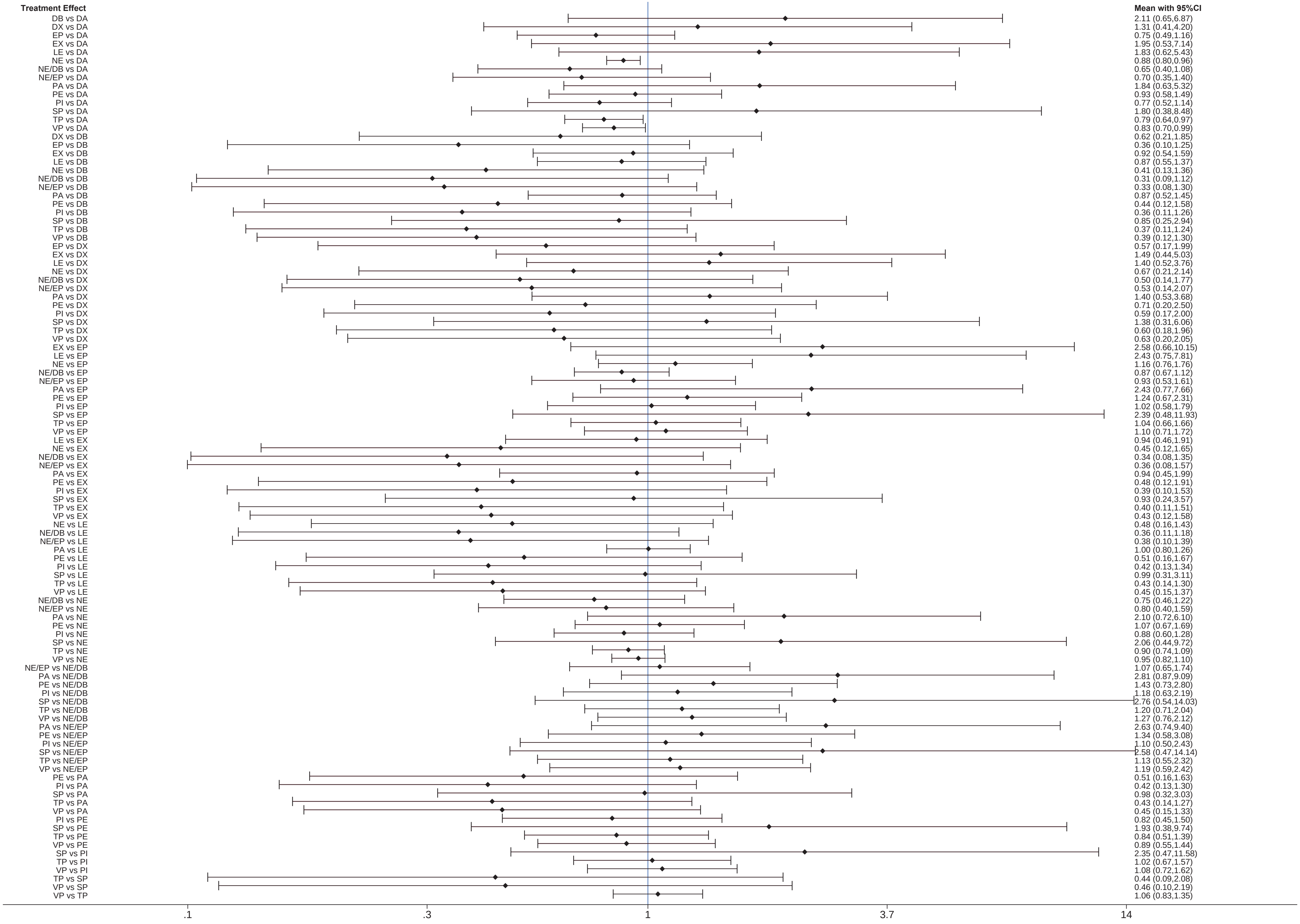

Supplementary Figure S2 Contribution plot for 28-day mortality

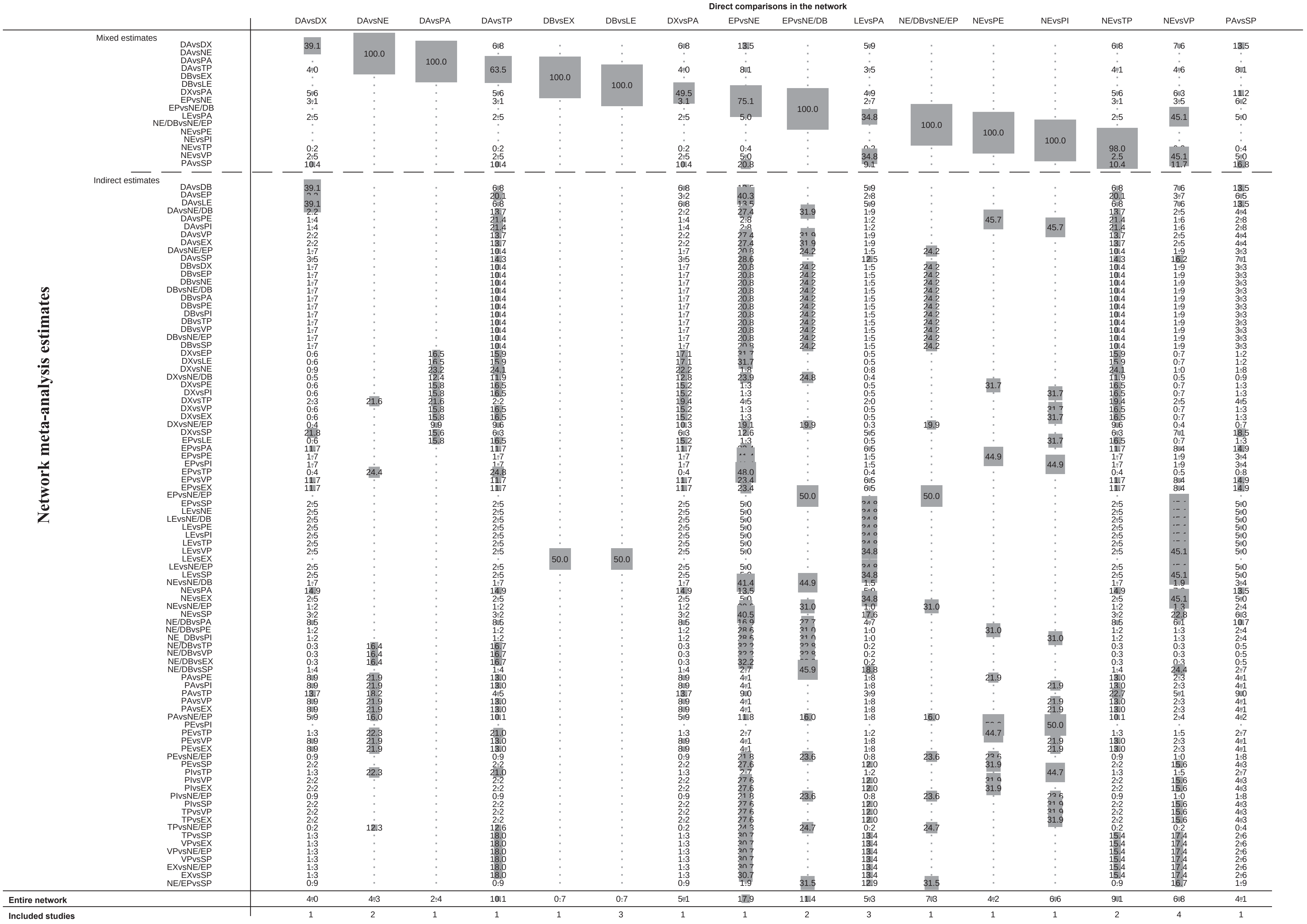

Supplementary Figure S3 Inconsistency analysis for 28-day mortality

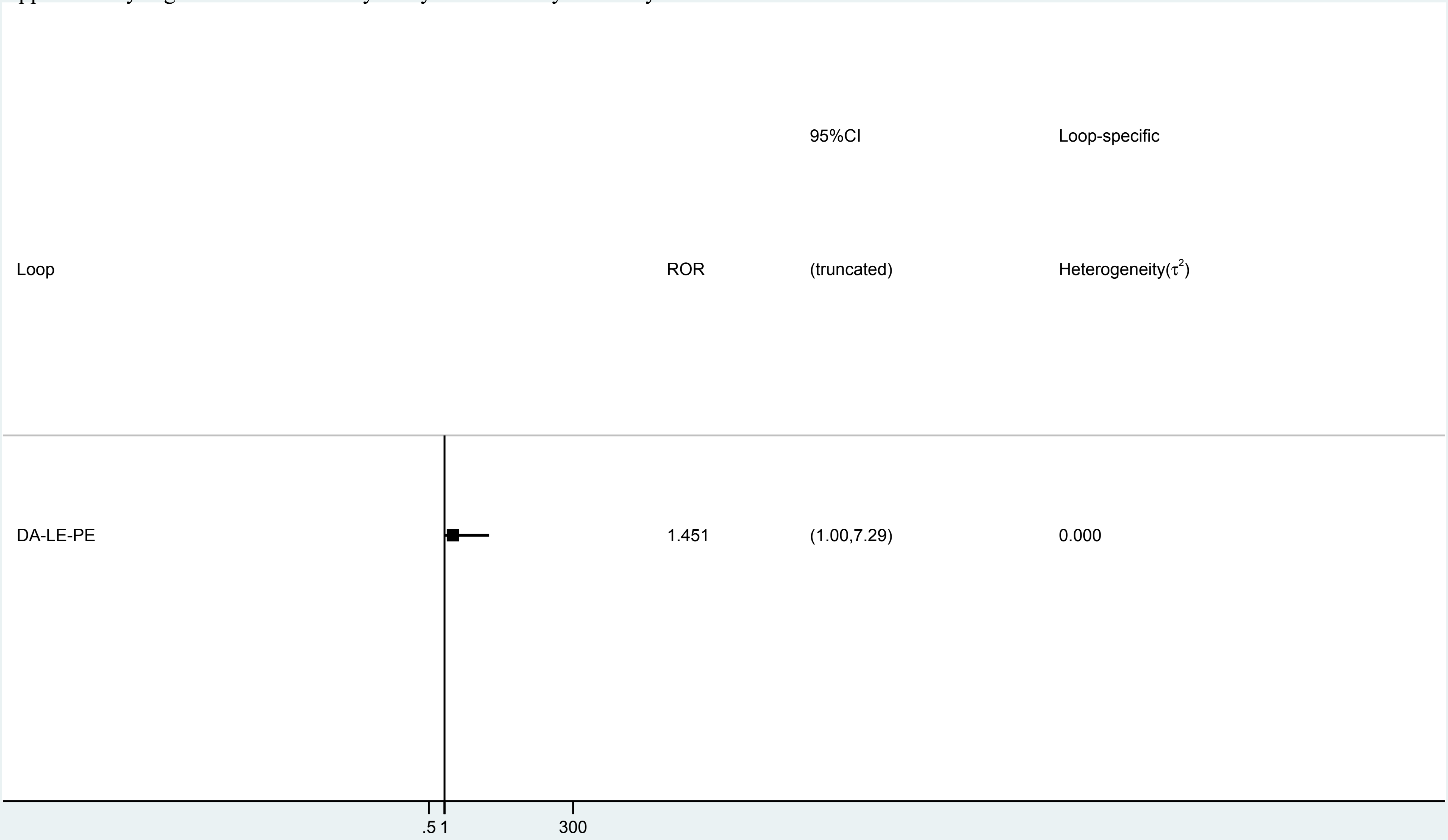

*** Loop(s) [DA-DX-NE/DB] are formed only by multi-arm trial(s) - Consistent by definition

Supplementary Figure S4 Network geometry for ICU mortality

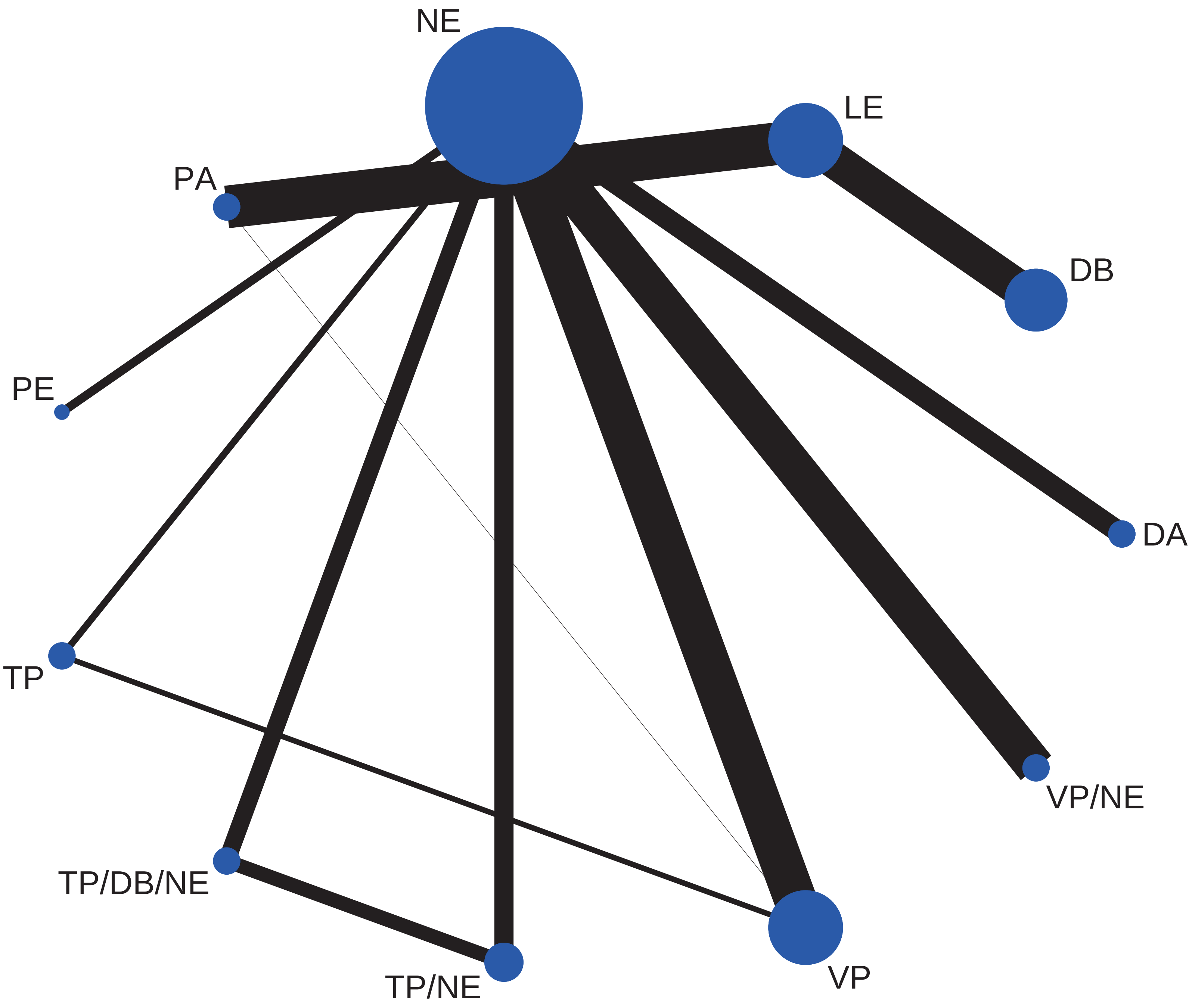

Supplementary Figure S5 SUCRA for ICU mortality

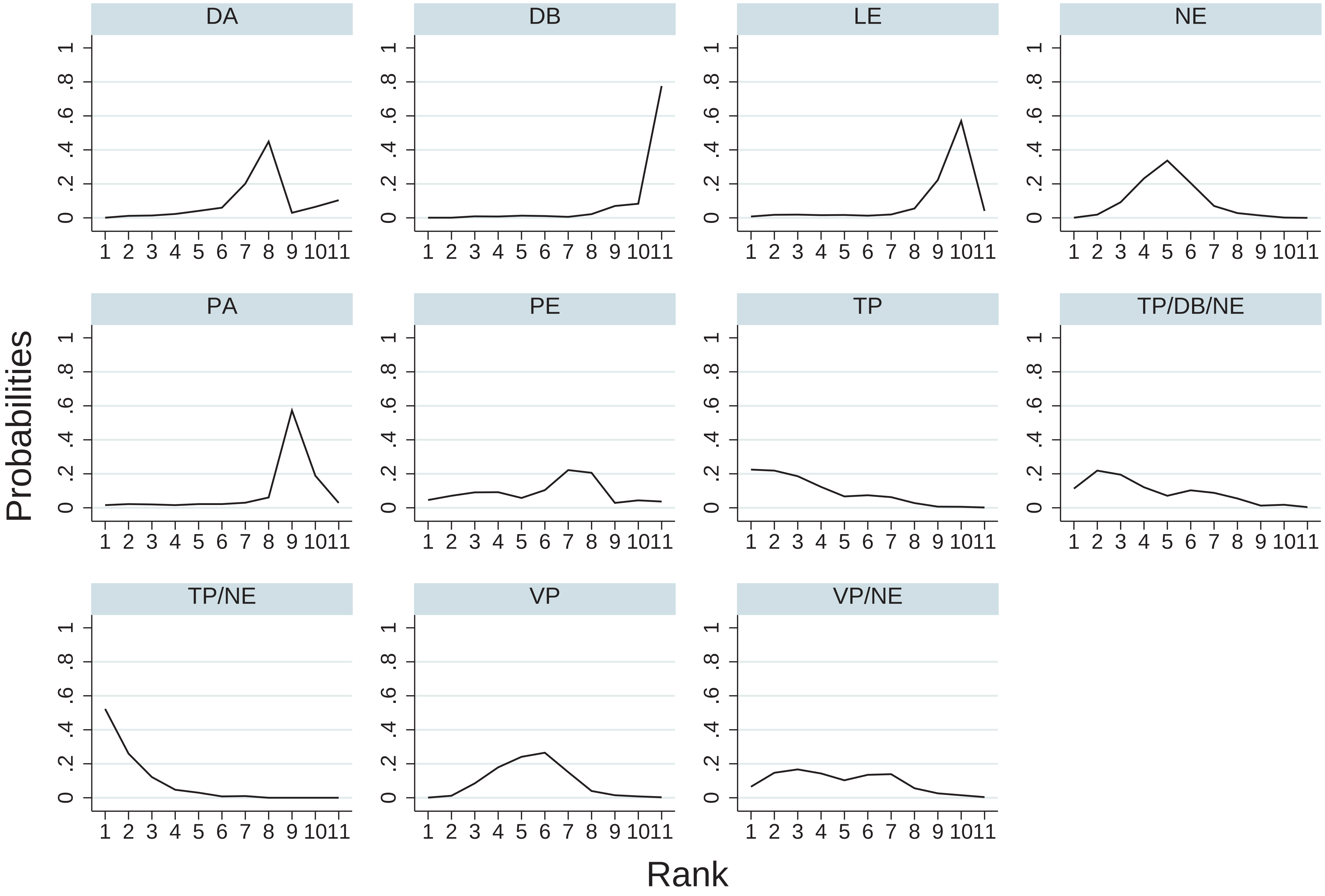

Supplementary Figure S6 Forest plot for ICU mortality

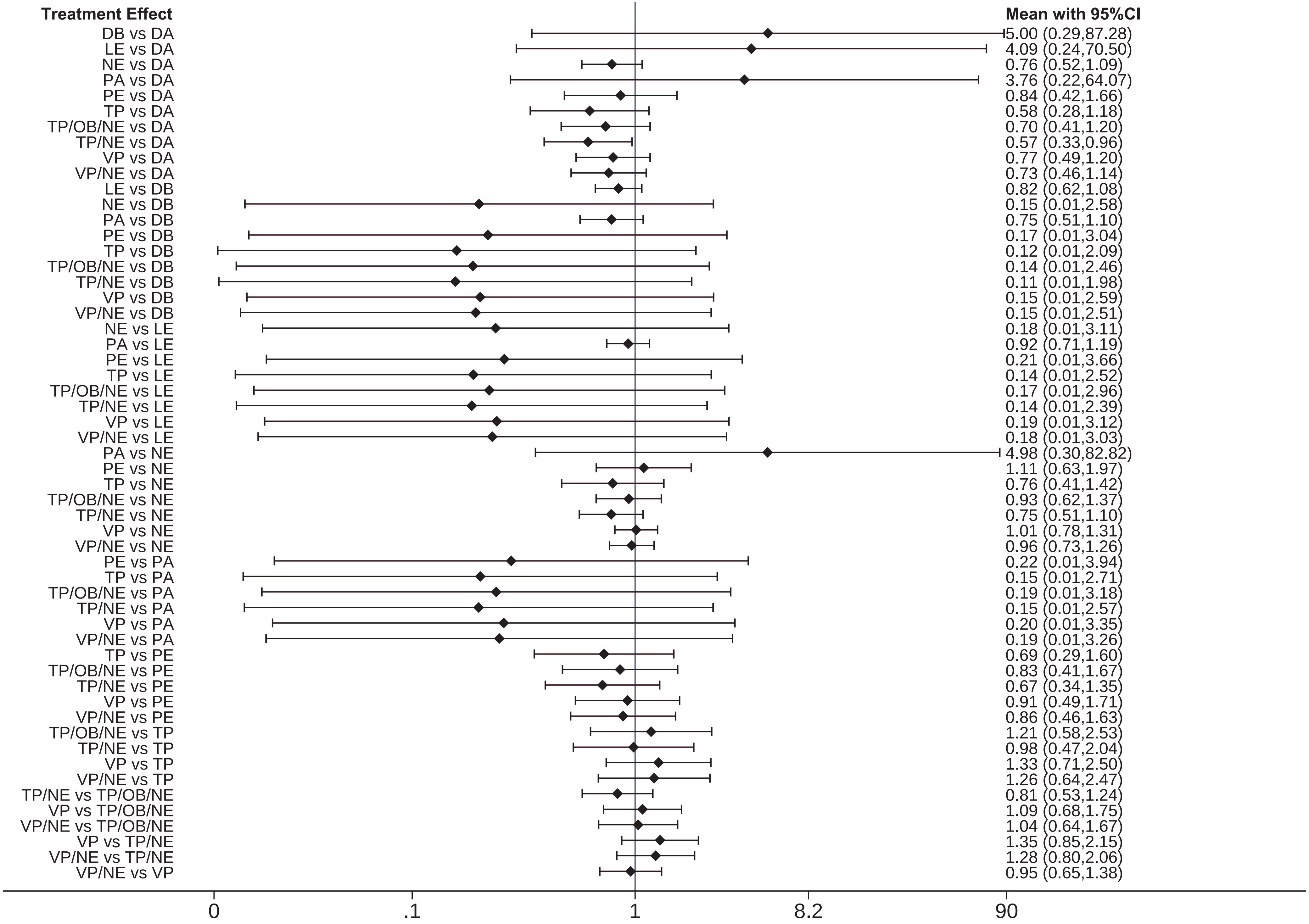

Supplementary Figure S7 Contribution plot for ICU mortality

Direct comparisons in the network

Network meta-analysis estimates

	DAvsNE	DBvsLE	LEvsPA	NEvsPE	NEvsTP	NEvsTP_DB_NE	NEvsTP_NE	NEvsVP	NEvsVP_NE	PAvsVP	TP_DB_NEvsTP_NE	TPvsVP
Mixed estimates												
DAvsNE	100.0	·	·	·	·	·	·	·	·	·	·	·
DBvsLE	·	100.0	·	·	·	·	·	·	·	·	·	·
LEvsPA	·	·	100.0	·	·	·	·	·	·	·	·	·
NEvsPE	·	·	·	100.0	·	·	·	·	·	·	·	·
NEvsTP	·	·	·	·	24.1	·	·	37.9	·	·	·	37.9
NEvsTP_DB_NE	·	·	·	·	·	75.4	12.3	·	·	·	12.3	·
NEvsTP_NE	·	·	·	·	·	29.5	41.0	·	·	·	29.5	·
NEvsVP	·	·	·	·	8.9	·	·	82.3	·	·	·	8.9
NEvsVP_NE	·	·	·	·	·	·	·	·	100.0	·	·	·
PAvsVP	0.1	0.2	0.3	0.1	·	0.1	·	0.3	0.1	98.7	·	0.1
TP_DB_NEvsTP_NE	·	·	·	·	·	30.6	30.6	·	·	·	38.8	·
TPvsVP	·	·	·	·	22.5	·	·	22.5	·	·	·	54.9
Indirect estimates												
DAvsDB	19.7	19.6	19.6	·	1.9	·	·	17.7	·	19.6	·	1.9
DAvsLE	24.4	·	24.4	·	2.4	·	·	22.0	·	24.3	·	2.4
DAvsPA	32.3	0.1	0.1	·	3.1	·	·	29.0	·	32.2	·	3.1
DAvsPE	50.0	·	·	50.0	·	·	·	·	·	·	·	·
DAvsTP	38.3	·	·	·	14.9	·	·	23.4	·	·	·	23.4
DAvsTP_DB_NE	46.7	·	·	·	·	40.2	6.6	·	·	·	6.6	·
DAvsTP_NE	41.3	·	·	·	·	17.3	24.0	·	·	·	17.3	·
DAvsVP	47.7	·	·	·	4.6	·	·	43.0	·	·	·	4.6
DAvsVP_NE	50.0	·	·	·	·	·	·	·	50.0	·	·	·
DBvsNE	·	24.4	24.4	·	2.4	·	·	22.0	·	24.4	·	2.4
DBvsPA	·	50.0	50.0	·	·	·	·	·	·	·	·	·
DBvsPE	·	19.6	19.6	19.7	1.9	·	·	17.7	·	19.6	·	1.9
DBvsTP	·	23.3	23.3	·	6.8	·	·	6.7	·	23.3	·	16.5
DBvsTP_DB_NE	·	19.1	19.1	·	1.9	16.4	2.7	17.2	·	19.0	2.7	1.8
DBvsTP_NE	·	18.1	18.1	·	1.8	7.6	10.6	16.3	·	18.1	7.6	1.8
DBvsVP	·	33.3	33.2	·	·	·	·	0.1	·	33.2	·	·
DBvsVP_NE	·	19.6	19.6	·	1.9	·	·	17.7	19.7	19.6	·	1.9
LEvsNE	·	0.1	32.3	·	3.1	·	·	29.1	·	32.2	·	3.1
LEvsPE	·	·	24.4	24.4	2.4	·	·	22.0	·	24.3	·	2.4
LEvsTP	·	0.1	30.4	·	8.9	·	·	8.8	·	30.3	·	21.6
LEvsTP_DB_NE	·	·	23.6	·	2.3	20.3	3.3	21.3	·	23.5	3.3	2.3
LEvsTP_NE	·	·	22.1	·	2.2	9.3	12.9	20.0	·	22.1	9.3	2.1
LEvsVP	·	0.1	49.8	·	·	·	·	0.2	·	49.7	·	·
LEvsVP_NE	·	·	24.4	·	2.4	·	·	22.0	24.4	24.3	·	2.4
NEvsPA	·	0.1	0.2	·	4.6	·	·	42.9	·	47.5	·	4.6
PAvsPE	·	0.1	0.1	32.3	3.1	·	·	29.0	·	32.2	·	3.1
PAvsTP	·	0.1	0.1	·	12.7	·	·	12.6	·	43.5	·	30.9
PAvsTP_DB_NE	·	0.1	0.1	·	3.0	26.5	4.3	27.8	·	30.8	4.3	3.0
PAvsTP_NE	·	·	0.1	·	2.8	11.9	16.5	25.6	·	28.3	11.9	2.7
PAvsVP_NE	·	0.1	0.1	·	3.1	·	·	29.0	32.3	32.2	·	3.1
PEvsTP	·	·	·	38.3	14.9	·	·	23.4	·	·	·	23.4
PEvsTP_DB_NE	·	·	·	46.7	·	40.2	6.6	·	·	·	6.6	·
PEvsTP_NE	·	·	·	41.3	·	17.3	24.0	·	·	·	17.3	·
PEvsVP	·	·	·	47.7	4.6	·	·	43.0	·	·	·	4.6
PEvsVP_NE	·	·	·	50.0	·	·	·	·	50.0	·	·	·
TPvsTP_DB_NE	·	·	·	·	14.1	31.2	5.1	22.2	·	·	5.1	22.2
TPvsTP_NE	·	·	·	·	12.8	13.8	19.2	20.2	·	·	13.8	20.2
TPvsVP_NE	·	·	·	·	14.9	·	·	23.4	38.3	·	·	23.4
TP_DB_NEvsVP	·	·	·	·	4.3	38.4	6.3	40.3	·	·	6.3	4.4
TP_DB_NEvsVP_NE	·	·	·	·	·	40.2	6.6	·	46.7	·	6.6	·
TP_NEvsVP	·	·	·	·	3.9	16.6	23.1	35.9	·	·	16.6	3.9
TP_NEvsVP_NE	·	·	·	·	·	17.3	24.0	·	41.3	·	17.3	·
VPvsVP_NE	·	·	·	·	4.6	·	·	43.0	47.7	·	·	4.6
**Entire network**	6.8	6.8	12.2	6.8	4.0	8.1	4.7	17.9	6.8	16.2	3.8	6.0
**Included studies**	2	5	1	1	1	1	2	4	2	1	1	1

Supplementary Figure S8 Inconsistency analysis for ICU mortality

Loop	IF	95%CI (truncated)	Loop-specific Heterogeneity(τ^2)
NE-TP/DB/NE-TP/NE	0.73	(0.00,1.70)	0.000
NE-TP-VP	0.29	(0.00,1.30)	0.000

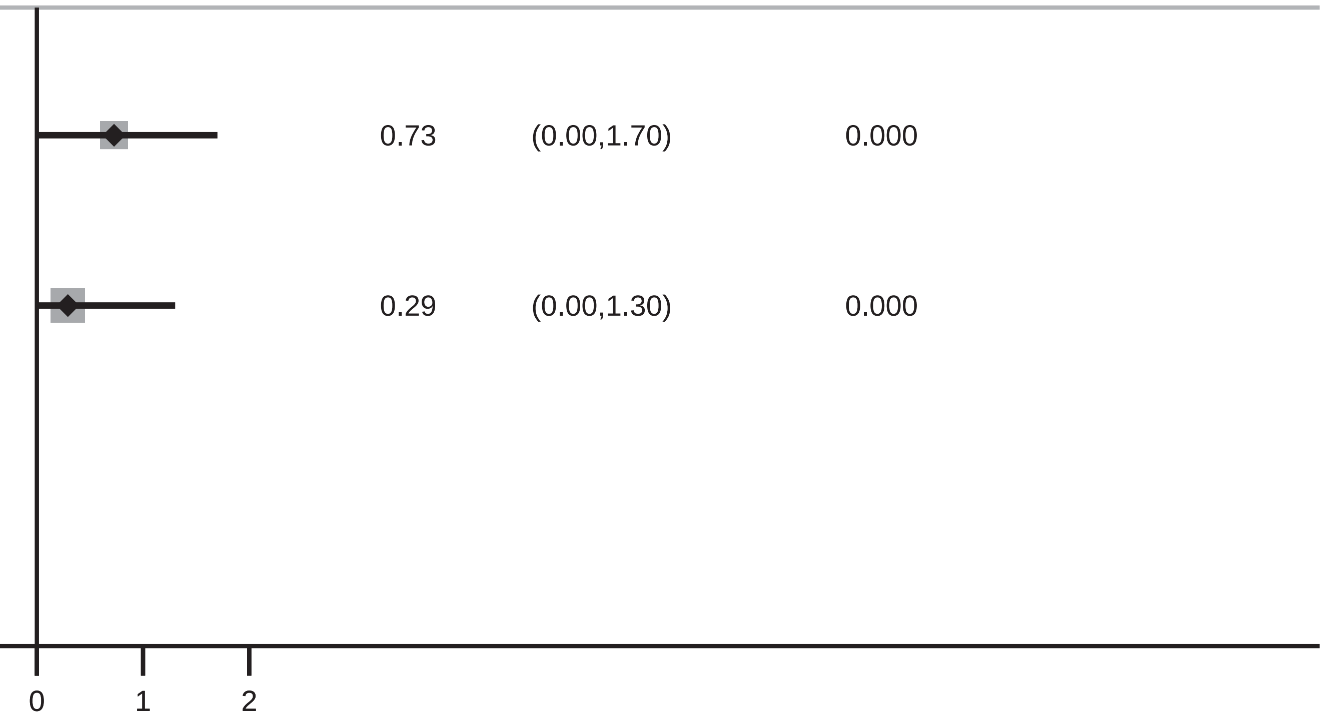

Supplementary Figure S9 Funnel plot for ICU mortality

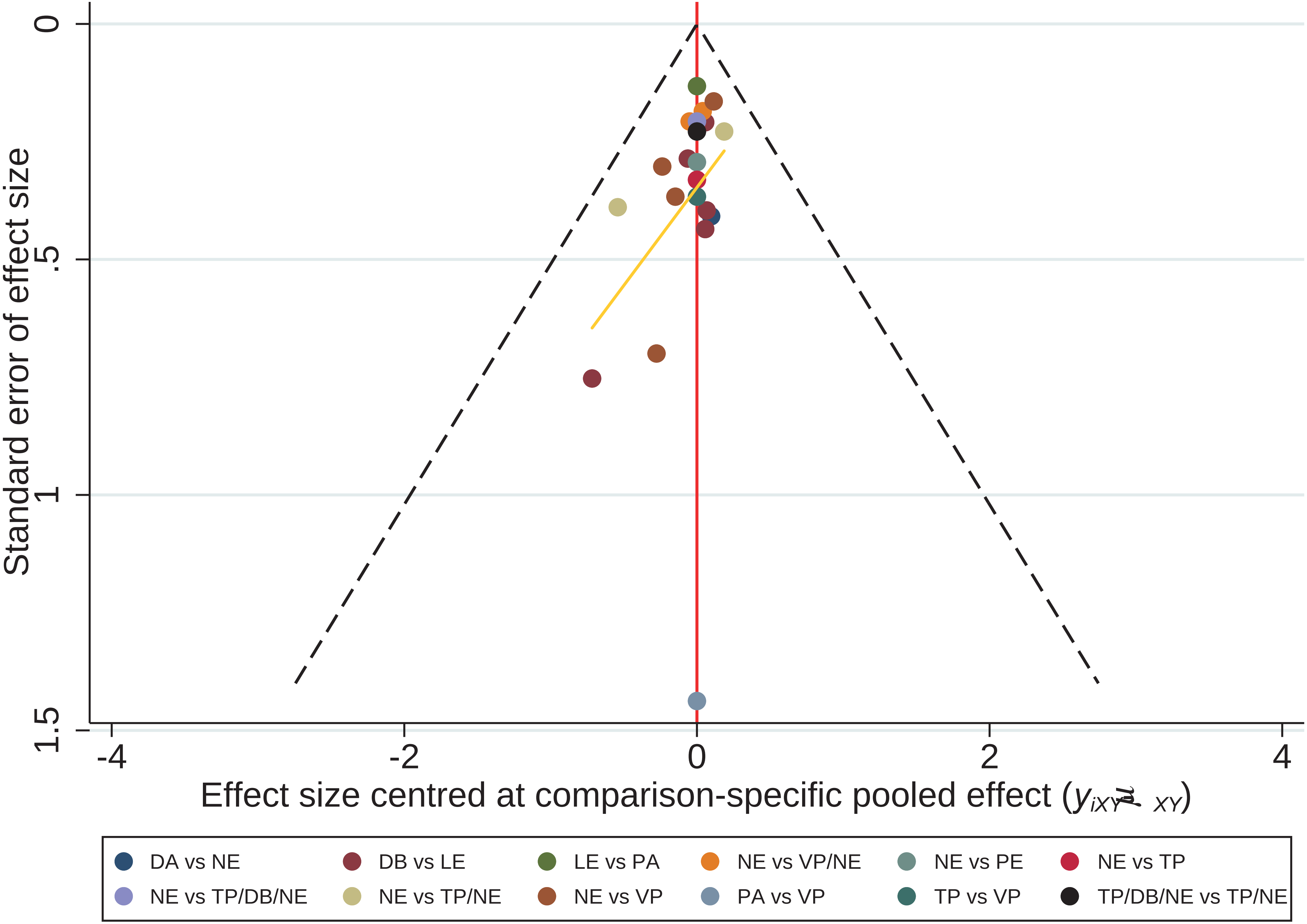

Supplementary Figure S10 Network geometry for ICU length of stay

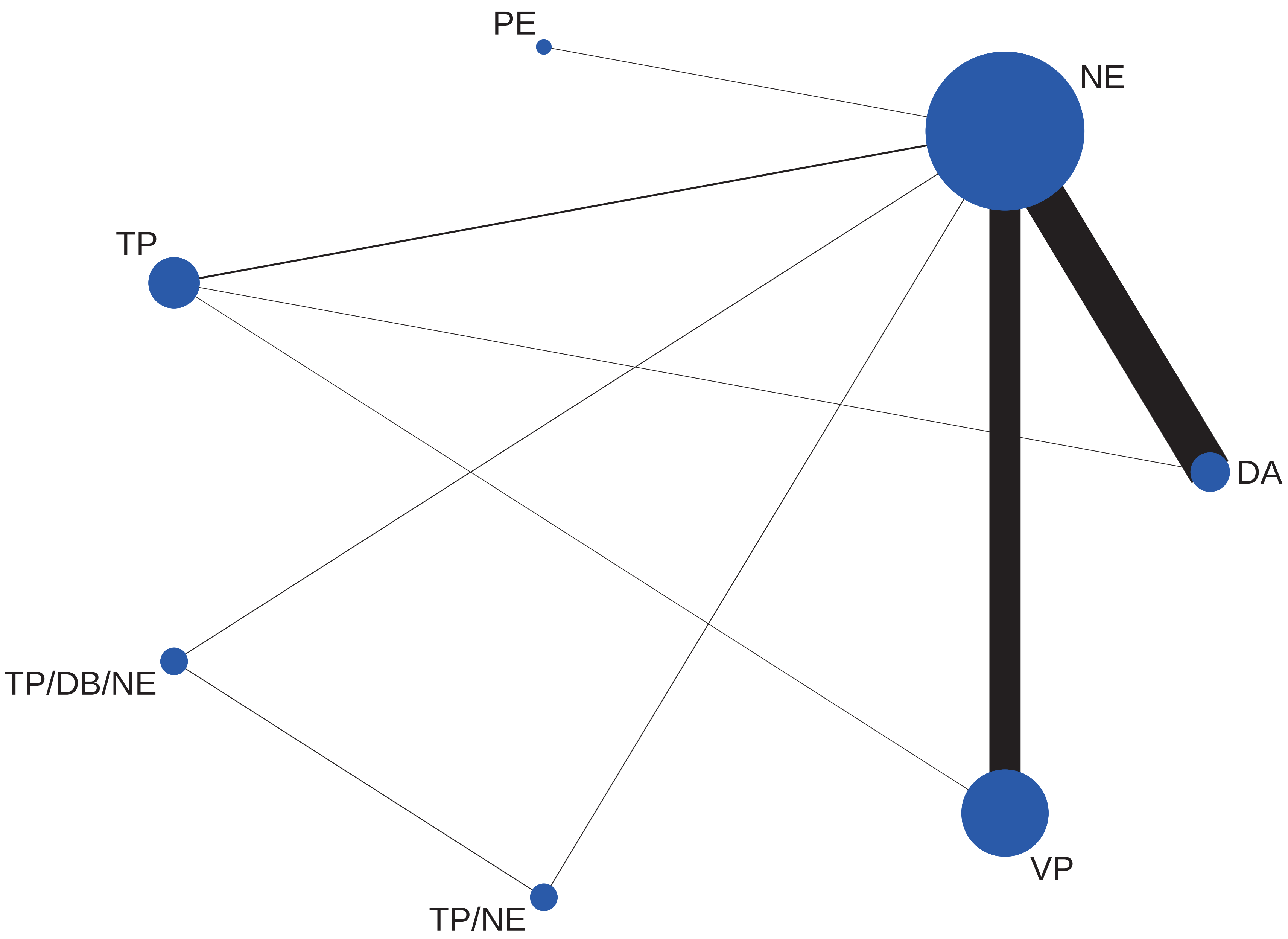

Supplementary Figure S11 SUCRA for ICU length of stay

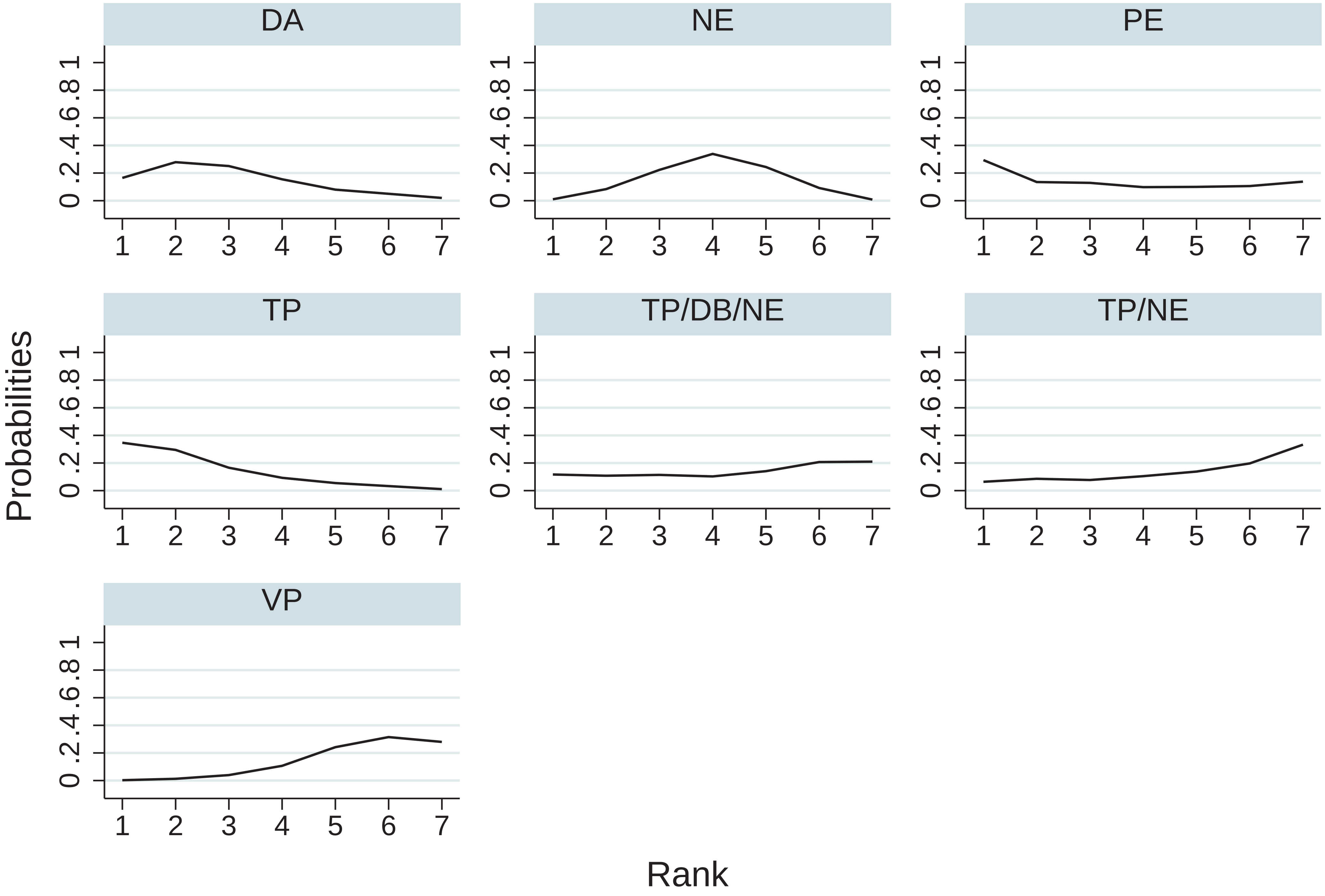

Supplementary Figure S12 Forest plot for ICU length of stay

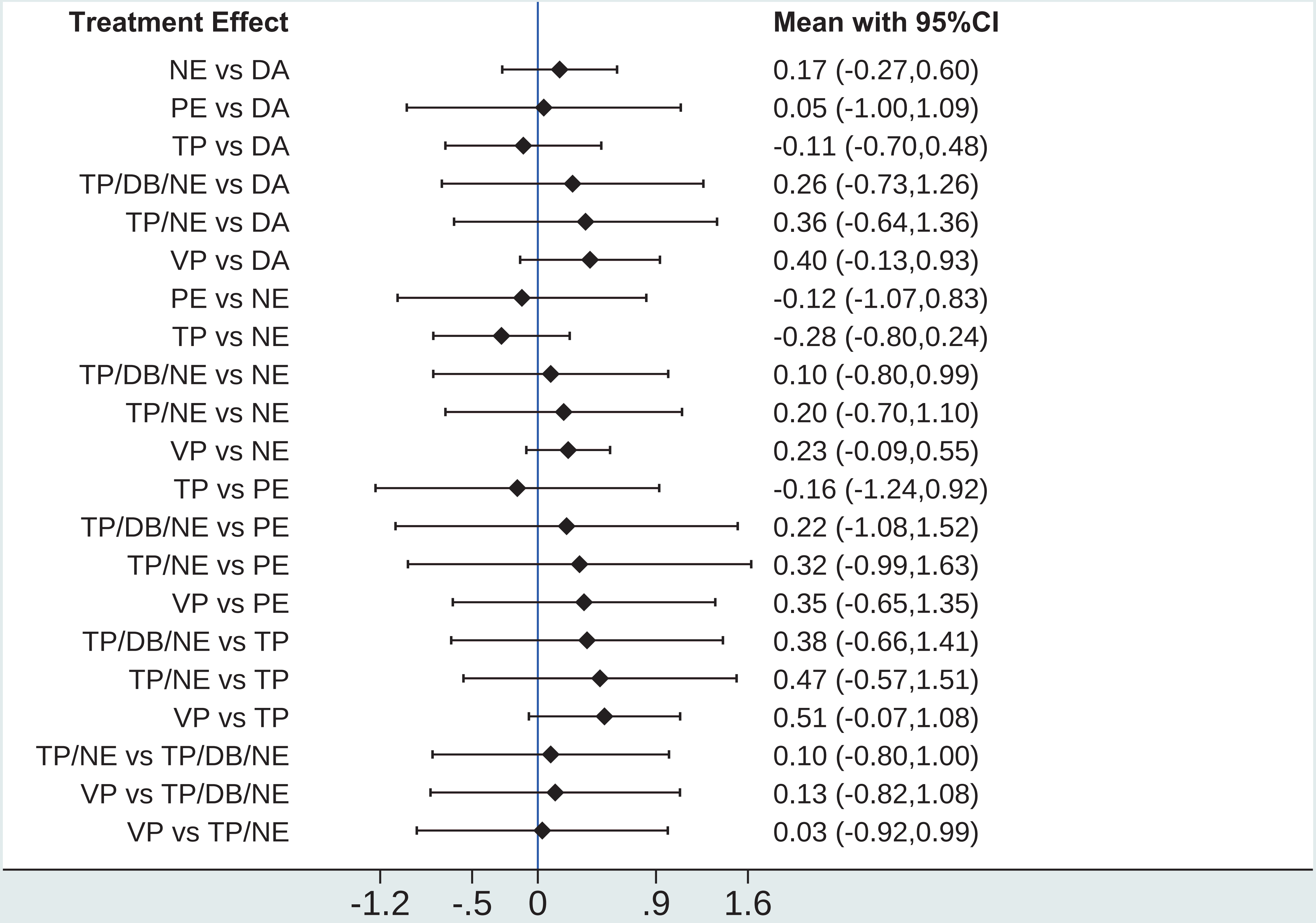

Supplementary Figure S13 Contribution plot for ICU length of stay

Direct comparisons in the network

Network meta-analysis estimates	DAvsNE	DAvsTP	NEvsPE	NEvsTP	NEvsTP/DB/NE	NEvsTP/NE	NEvsVP	TP/DB/NEvsTP/NE	TPvsVP
**Mixed estimates**									
DAvsNE	89.1	4.9	·	3.7	·	·	1.1	·	1.1
DAvsTP	38.6	13.8	·	29.7	·	·	9.0	·	9.0
NEvsPE	·	·	100.0	·	·	·	·	·	·
NEvsTP	15.1	15.1	·	43.5	·	·	13.1	·	13.1
NEvsTP/DB/NE	·	·	·	·	54.2	22.9	·	22.9	·
NEvsTP_NE	·	·	·	·	23.4	53.2	·	23.4	·
NEvsVP	5.3	5.3	·	15.3	·	·	53.6	·	20.6
TP/DB/NEvsTP/NE	·	·	·	·	28.4	28.4	·	43.1	·
TPvsVP	8.3	8.3	·	23.9	·	·	32.1	·	27.4
**Indirect estimates**									
DAvsPE	45.9	2.5	48.4	1.9	·	·	0.6	·	0.6
DAvsTP/DB/NE	40.2	2.2	·	1.7	29.8	12.6	0.5	12.6	0.5
DAvsTP/NE	40.0	2.2	·	1.7	12.9	29.3	0.5	12.9	0.5
DAvsVP	40.5	5.7	·	7.7	·	·	32.8	·	13.4
PEvsTP	8.8	8.8	41.8	25.3	·	·	7.6	·	7.6
PEvsTP/DB/NE	·	·	43.5	·	30.6	12.9	·	12.9	·
PEvsTP/NE	·	·	43.4	·	13.3	30.1	·	13.3	·
PEvsVP	3.0	3.0	42.6	8.8	·	·	30.8	·	11.8
TPvsTP/DB/NE	7.8	7.8	·	22.5	26.1	11.0	6.8	11.0	6.8
TPvsTP/NE	7.8	7.8	·	22.5	11.3	25.7	6.8	11.3	6.8
TP/DB/NEvsVP	2.7	2.7	·	7.8	26.6	11.2	27.3	11.2	10.5
TP/NEvsVP	2.7	2.7	·	7.8	11.5	26.2	27.2	11.5	10.5
**Entire network**	16.1	4.5	14.5	11.0	13.1	12.9	12.3	8.7	6.8
**Included studies**	2	1	1	2	1	1	6	1	1

Supplementary Figure S14 Inconsistency analysis for ICU length of stay

Loop		IF	95%CI (truncated)	Loop-specific Heterogeneity(τ^2)
NE-TP-VP		0.35	(0.00,1.91)	0.260
DA-NE-TP		0.19	(0.00,1.03)	0.005

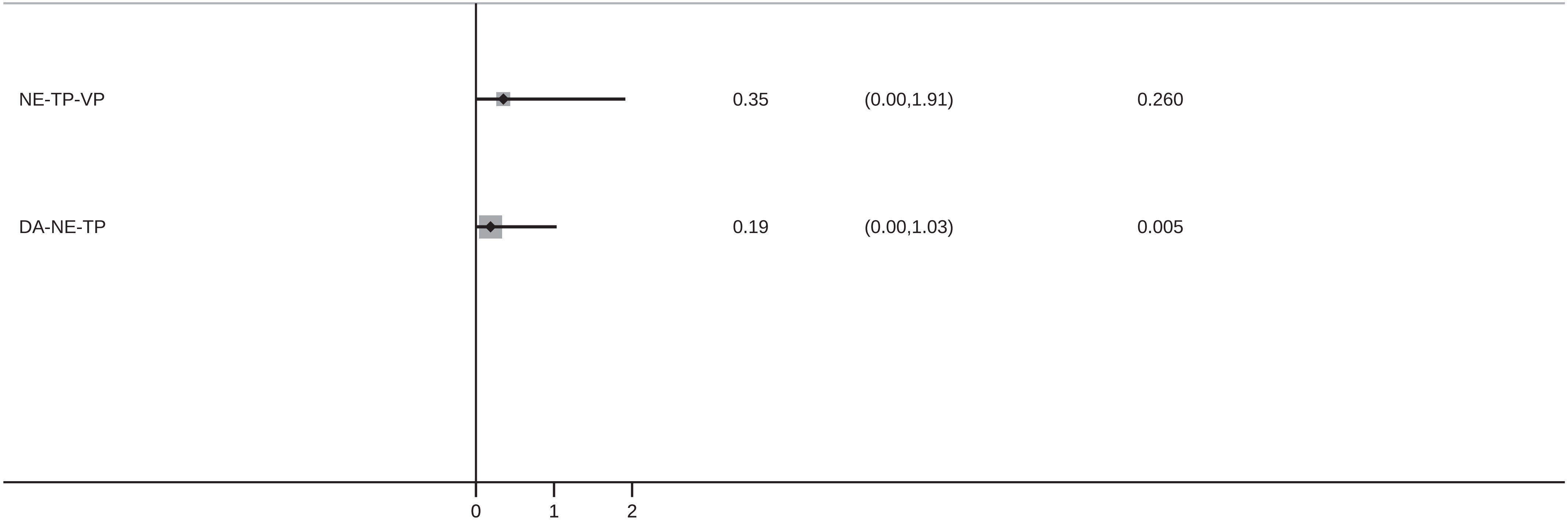

*** Loop(s) [NE-TP/DB/NE-TP/NE] are formed only by multi-arm trial(s) - Consistent by definition

Supplementary Figure S15 Funnel plot for ICU length of stay

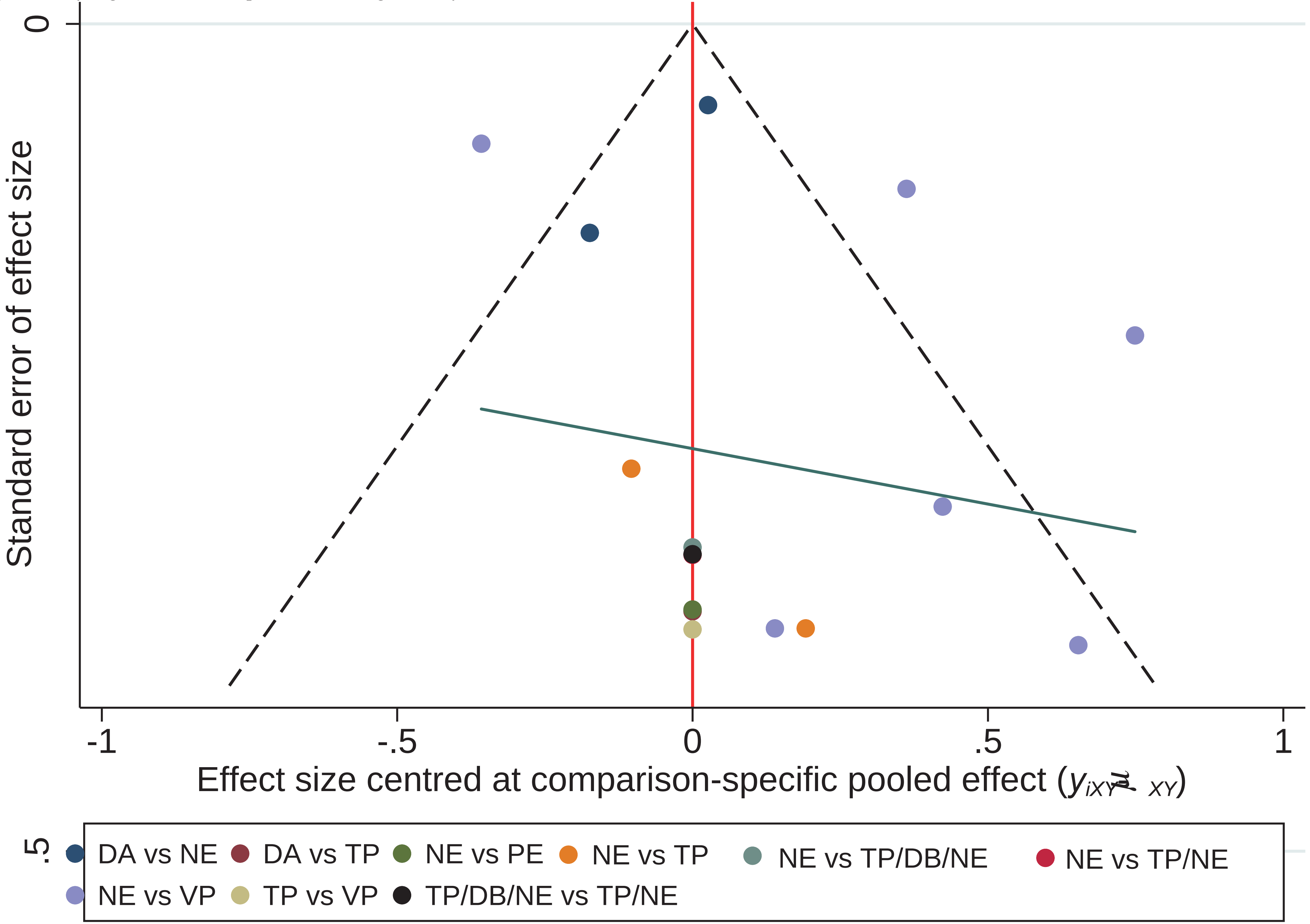

Supplementary Figure S16 Network geometry for hospital length of stay

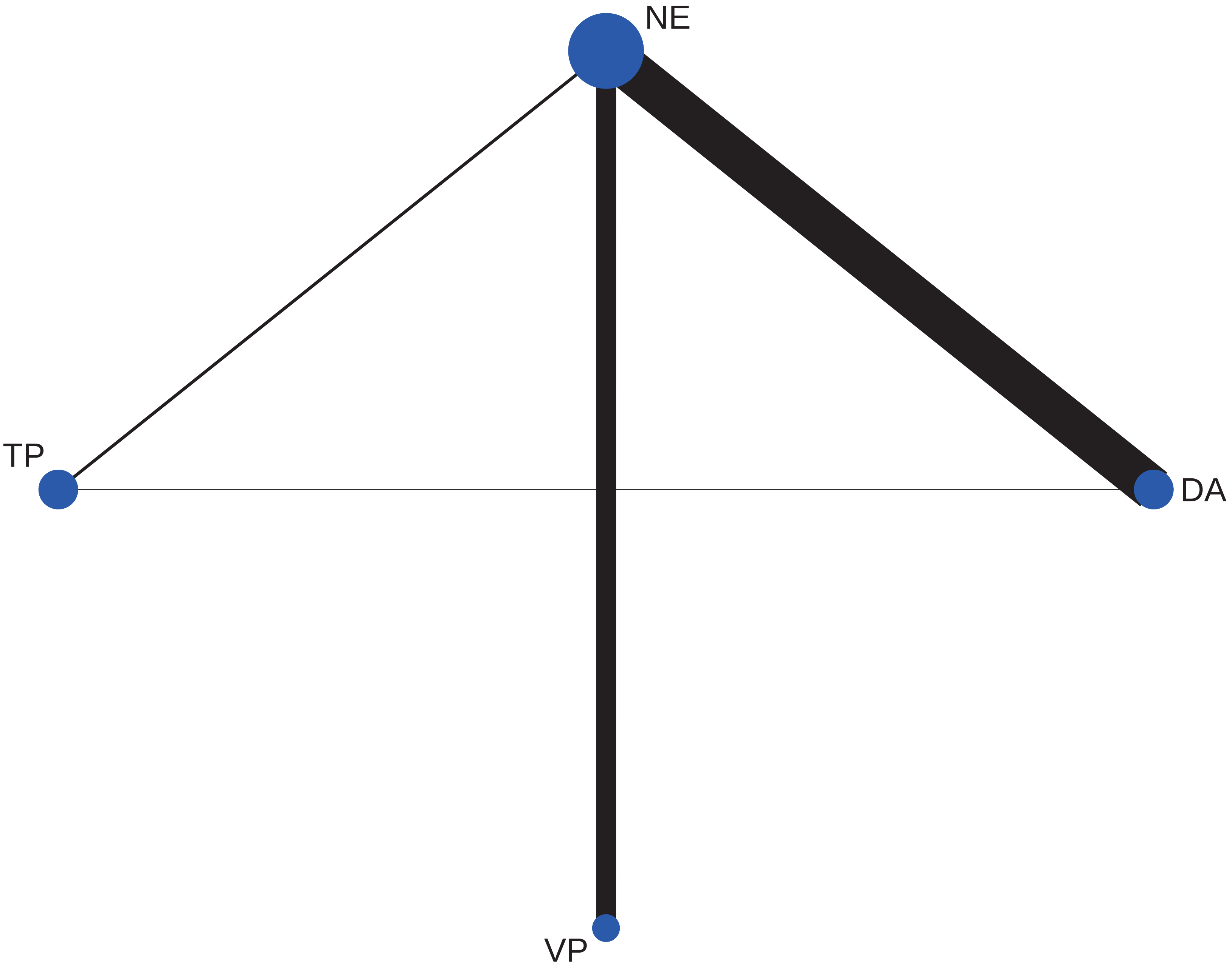

Supplementary Figure S17 SUCRA for hospital length of stay

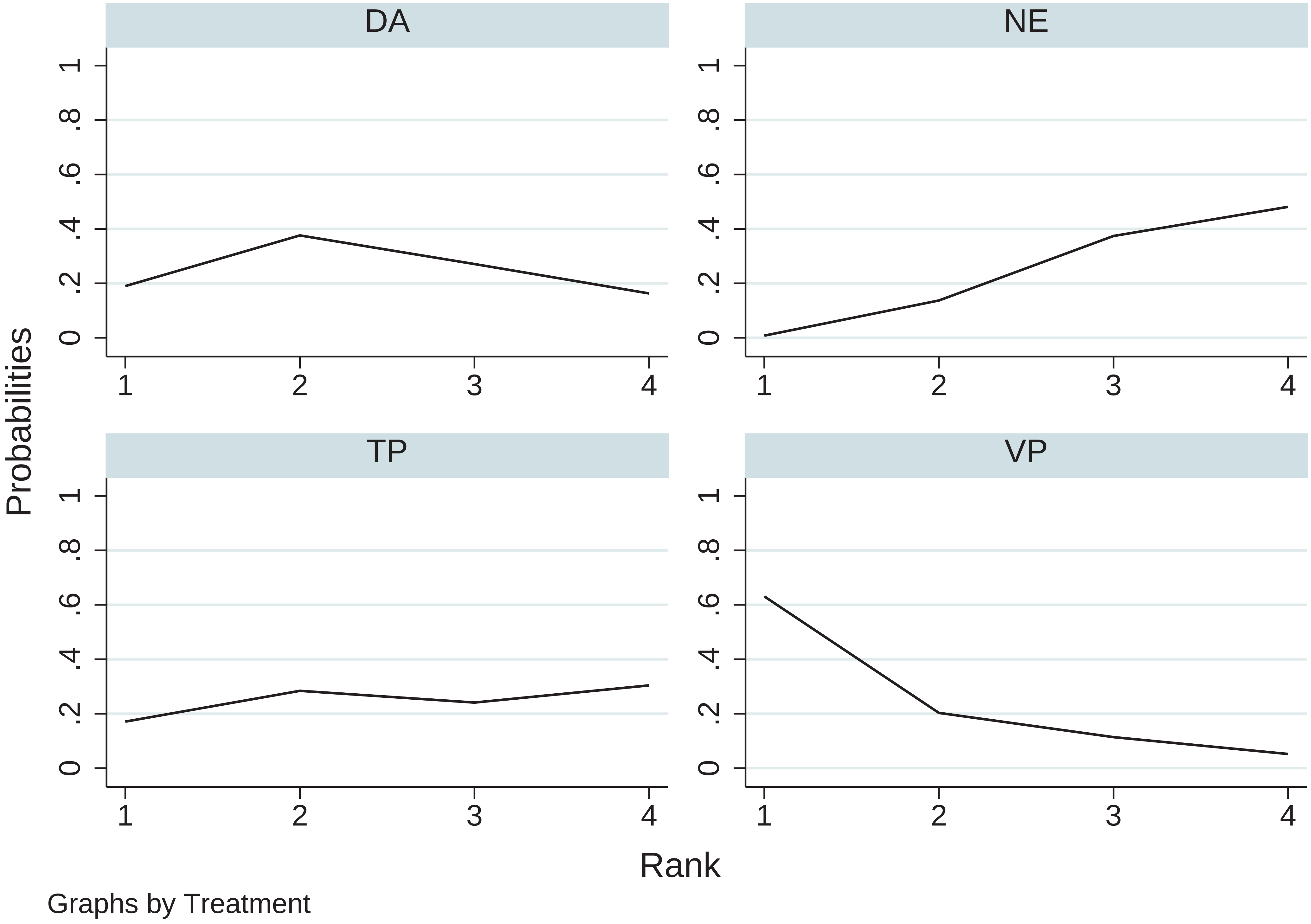

Supplementary Figure S18 Forest plot for hospital length of stay

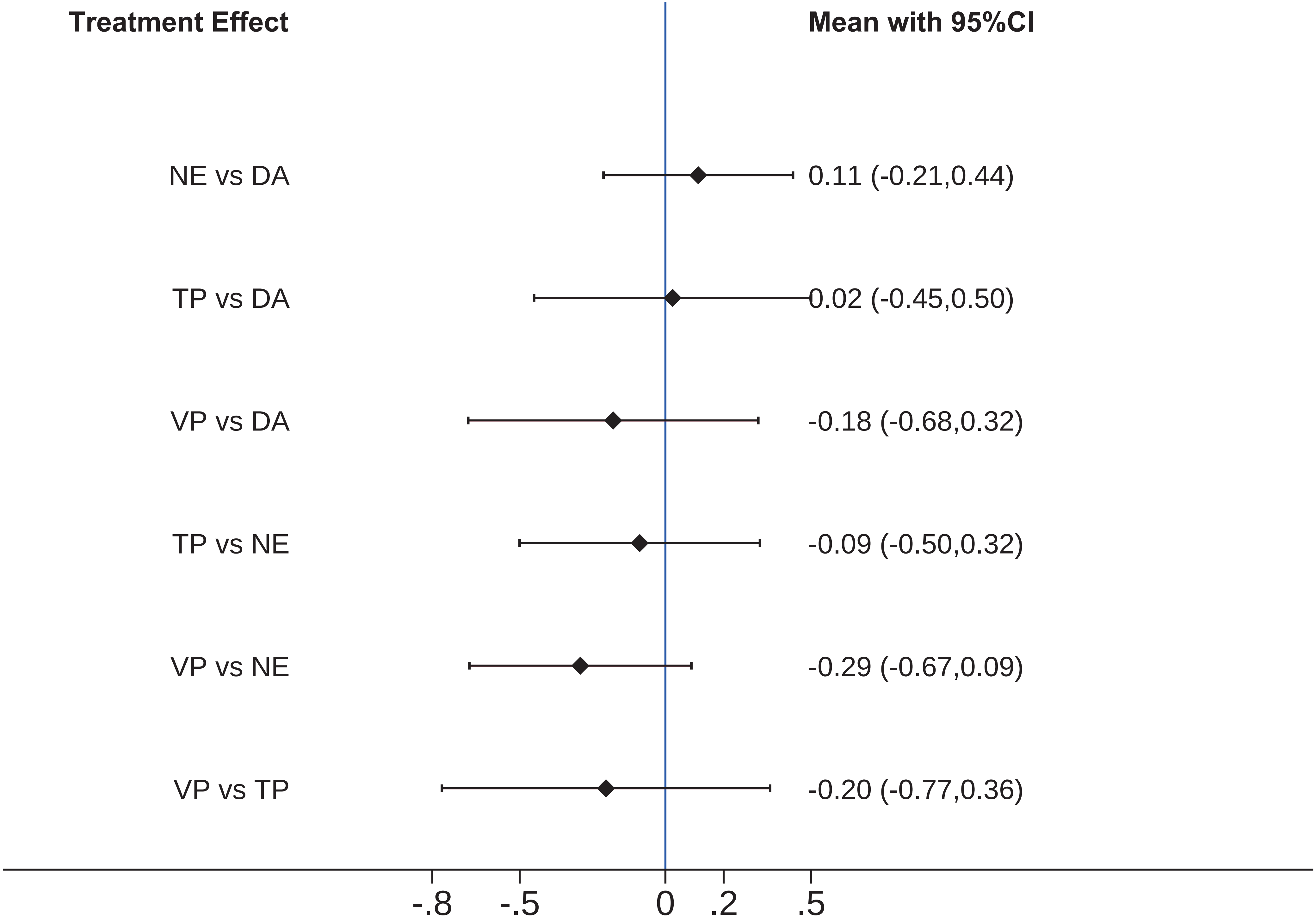

Supplementary Figure S19 Contribution plot for hospital length of stay

Direct comparisons in the network

Network meta-analysis estimates		DAvsNE	DAvsTP	NEvsTP	NEvsVP
Mixed estimates					
	DAvsNE	87.0	6.5	6.5	·
	DAvsTP	43.1	13.7	43.1	·
	NEvsTP	14.7	14.7	70.6	·
	NEvsVP	·	·	·	100.0
Indirect estimates					
	DAvsVP	45.0	3.3	3.3	48.3
	TPvsVP	7.9	7.9	38.1	46.0
**Entire network**		32.1	7.8	27.6	32.5
**Included studies**		2	1	2	2

Supplementary Figure S20 Inconsistency analysis for hospital length of stay

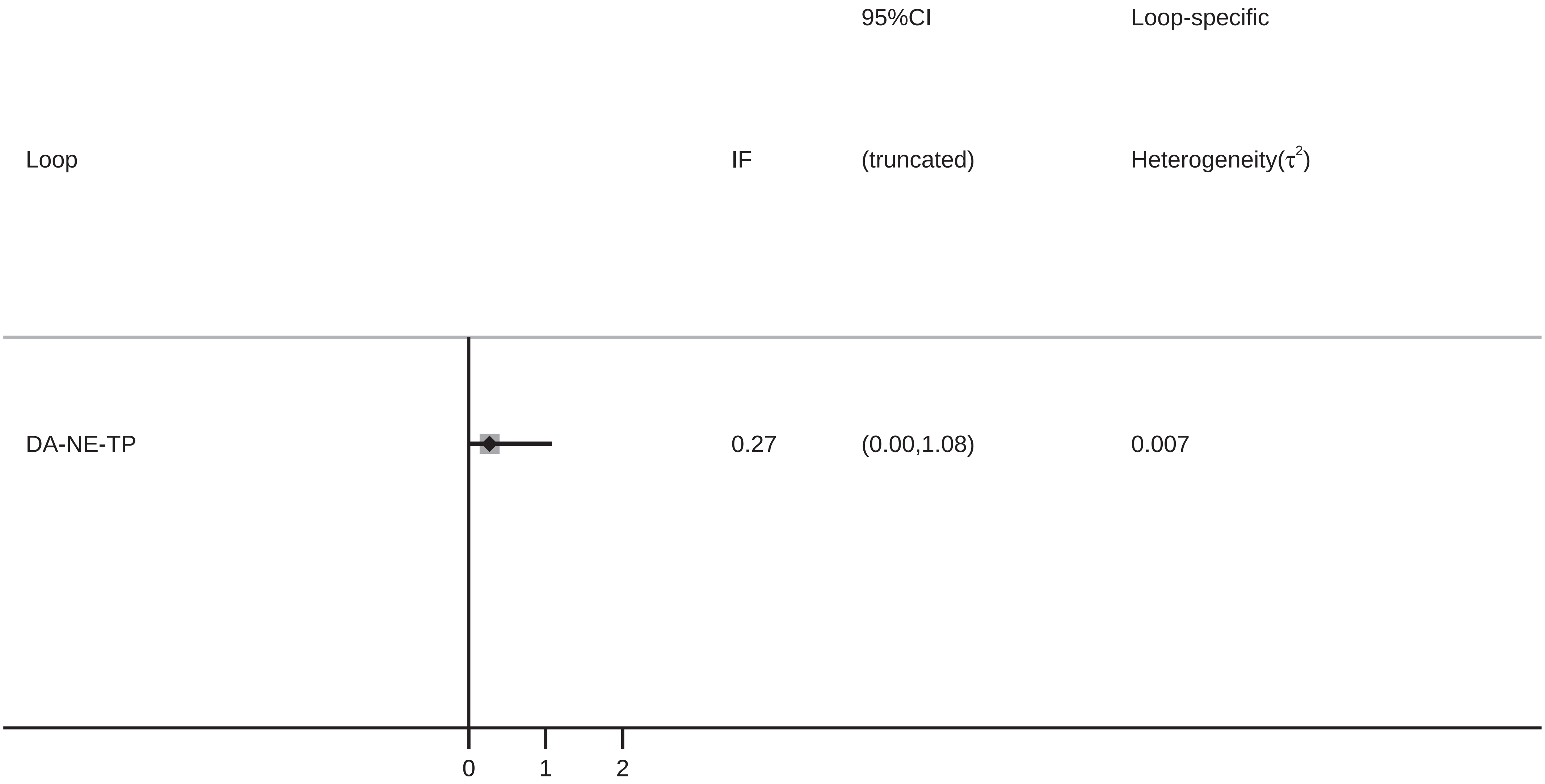

Supplementary Figure S21 Funnel plot for hospital length of stay

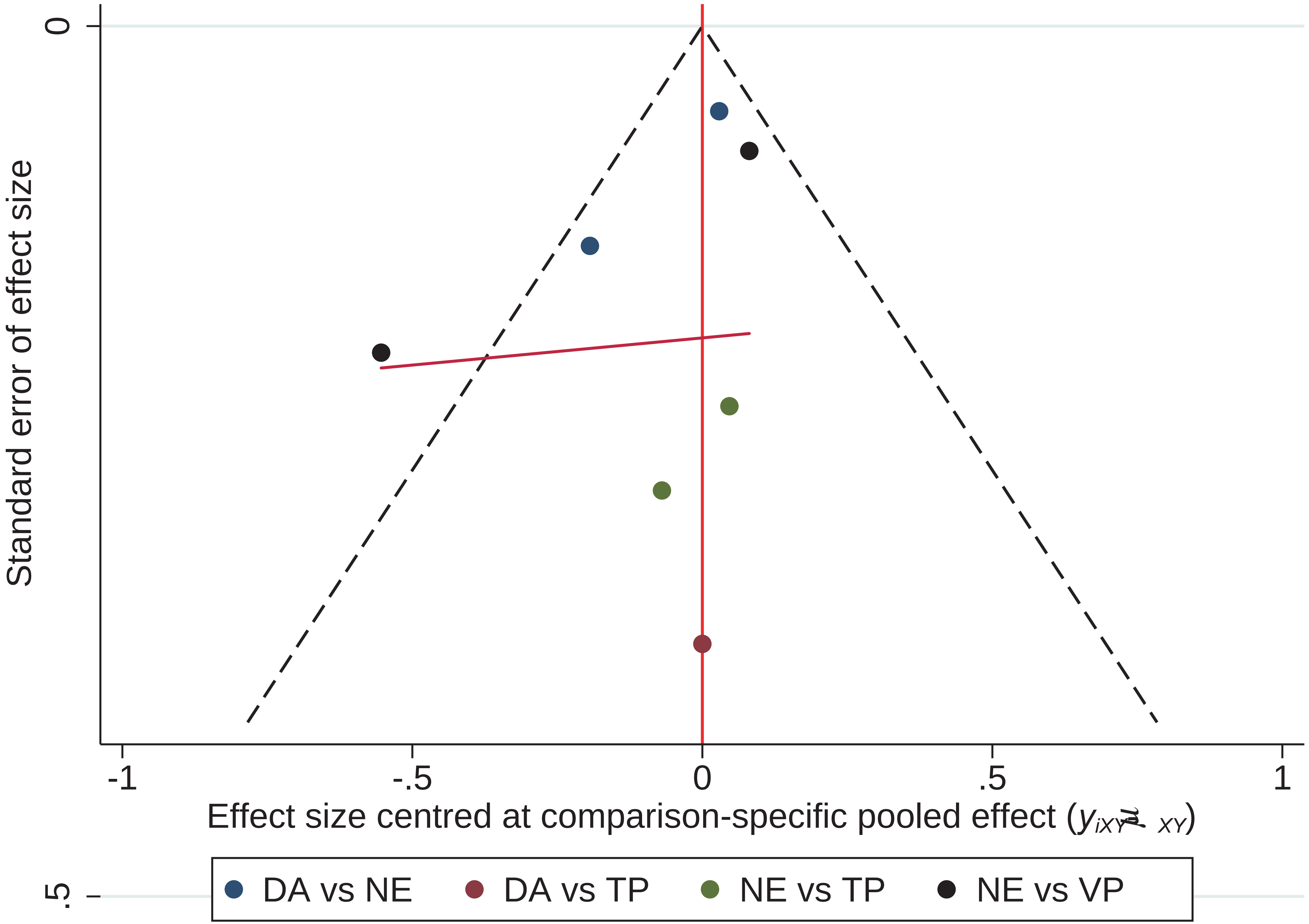

Supplement: Supplementary file 1 — Table S1. Study characteristics: management description of included randomized controlled trials. Table S2. Assessment of risk of bias. Figure S1-S3. Forest plot for the overall comparisons, contribution plot, and inconsistency analysis for 28-day mortality. Figure S4-S9. Network geometry, SUCRA, forest plot, contribution plot, inconsistency analysis, and funnel plot for ICU mortality. Figure S10-S15. Network geometry, SUCRA, forest plot, contribution plot, inconsistency analysis, and funnel plot for ICU length of stay. Figure S16-S21. Network geometry, SUCRA, forest plot, contribution plot, inconsistency analysis, and funnel plot for hospital length of stay. (PDF 1411 kb) [file 13054_2019_2427_MOESM1_ESM.pdf]
